# A biochemical difference between HAI-2 variants causing syndromic and non-syndromic Congenital Sodium Diarrhea

**DOI:** 10.1371/journal.pone.0358119

**Published:** 2026-09-15

**Authors:** Maiken Jaller Jensen, Monika Mrackova, Annika Weile Nonboe, Emilie Tina Lembke, Christine Schar, Jan Kristian Jensen, Asli Silahtaroglu, René Villadsen, Lotte Katrine Vogel

**Affiliations:** 1 Department of Cellular and Molecular Medicine, Faculty of Health and Medical Sciences, University of Copenhagen, Copenhagen N, Denmark; 2 Department of Molecular Biology and Genetics, Aarhus University, Aarhus, Denmark; Public Library of Science, UNITED KINGDOM OF GREAT BRITAIN AND NORTHERN IRELAND

## Abstract

Variants in the *SPINT2* gene, encoding HAI-2, cause the syndromic form of autosomal recessive Congenital Sodium Diarrhea (CSD), a life-threatening disease with early-onset persistent diarrhea and stools containing elevated levels of sodium. A newly discovered variant, HAI-2 Y68C, causes a milder non-syndromic CSD-like condition in an isolated case. The present investigation compares biochemical features of the HAI-2 Y68C with two newly discovered syndromic CSD-causing variants, HAI-2 Y129C and R148H, and two previously characterized syndromic CSD-causing variants, HAI-2 F161V and G168S. HAI-2 contains two Kunitz domains that inhibit a range of serine proteases, including matriptase and prostasin. HAI-2 is co-located with target proteases mainly in the ER and the early secretory pathway but also on the apical plasma membrane of polarized cells. All investigated variants of HAI-2 were expressed with a similar steady-state protein level and weak pericellular staining as wild-type HAI-2. However, none of the CSD-causing variants were capable of inhibiting prostasin as efficiently as wild-type HAI-2. In addition, the HAI-2 Y68C variant was unable to inhibit matriptase, constituting a clear biochemical difference between the single variant causing non-syndromic CSD and the variants causing syndromic CSD. The HAI-2 Y68C variant may affect the phenotype by altering matriptase catalyzed activation of prostasin in the early secretory pathway. We hypothesize that CSD, whether syndromic or non-syndromic, is caused by a lack of inhibitory activity of HAI-2 in the early secretory pathway and/or on the apical plasma membrane, resulting in uncontrolled proteolytic activity leading to proteolytic modification of sodium transporters/channels like NHE3 and ENaC. NHE3 and ENaC are normally located on the apical plasma membrane and are mainly responsible for the uptake of sodium from the gut lumen. Oral administration of a protease inhibitor mimicking HAI-2 wild-type could potentially help to reduce the degradation of proteins important for sodium uptake across the apical plasma membrane.

## Introduction

Congenital Sodium Diarrhea (CSD) is a rare disorder, which in many cases is accompanied by a shortened life expectancy. The disorder is characterized by intractable secretory diarrhea with an elevated concentration of sodium in the stool [1]. Although CSD is heterogeneous both clinically and genetically, it is common to distinguish between a non-syndromic and a syndromic form [2]. The non-syndromic CSD patients only display symptoms related to persistent diarrhea [2]. Non-syndromic CSD may be caused by recessive loss-of-function mutations in the *SLC9A3* gene, which encodes the sodium-hydrogen exchanger 3 (NHE3), or from dominant gain-of-function mutations in the *GUCY2C* gene, which encodes guanylate cyclase C (GC-C) [3,4]. The protein product of the CSD-causing variants of GC-C leads to inhibition of NHE3 [4,5]. The NHE3 antiporter is located on the apical plasma membrane of enterocytes, where it contributes to the uptake of sodium from the intestinal lumen [6].

Patients suffering from syndromic CSD often carry mutation in the *SPINT2* gene encoding the serine protease inhibitor hepatocyte growth factor activator inhibitor 2 (HAI-2). Syndromic CSD patients display additional malformations, often including choanal atresia and corneal erosions/superficial punctate keratitis [2,7] or, less frequently, intestinal atresia, hypertelorism, long philtrum, short and brittle hair/pilar dysplasia, cleft lip and palate, bone malformations, polydactyly and optic coloboma [2,7–13]. Some syndromic CSD patients also present with intestinal tufts upon histologic examination and consequently, syndromic CSD has also been referred to as a syndromic form of congenital tufting enteropathy [7,10,12].

According to Genome Aggregation Database (GnomAD version 4.1.0) 276 missense and 19 frameshift or splice site variants have been reported for the *SPINT2* gene. In total,16 different *SPINT2* variants have been reported to cause syndromic CSD in a homozygous or compound heterozygous state [2,7–10,13–15] including six missense variants: Y129C [14], R148C [7], R148H [13], F161V [9], Y163C [2] and G168S [7,9]. The HAI-2 variants causing CSD are extremely rare (Table 1) with the HAI-2 Y163C variant as the most frequent. Consequently, almost all children born with this condition are born from consanguineous parents.

**Table 1:** Frequency of HAI-2 missense variants analyzed in the present study.

HAI-2 variant	Abbreviation	SNV	Reference	Genomic location (GRCh38)	Reference SNP cluster ID	Refseq Accession	Allele frequency (GnomAD)	Clinvar ID
**p.Tyr68Cys**	Y68C	c.203A > G	[15]	chr19: 38283723	NA	NM_021102.4:c.203A > G	0.0000048	NA
**p.Tyr129Cys**	Y129C	c.386A > G	[14]	chr19: 38289186	rs2146278813	NM_021102.4:c.386A > G	NA	1321871
**p.Arg148His**	R148H	c.443G > A	[13]	chr19: 38290170	rs1353175955	NM_021102.4:c.443G > A	0.0000031	NA
**p.Phe161Val**	F161V	c.481T > G	[9]	chr19: 38290208	NA	NM_021102.4:c.481T > G	NA	NA
**p.Tyr163Cys**	Y163C	c.488A > G	[2]	chr19: 38290215	rs121908403	NM_021102.4:c.488A > G	0.00019	5205
**p.Gly168Ser**	G168S	c.502G > A	[7]	chr19: 38290229	rs606231284	NM_021102.4:c.502G > A	0.0000080	15707

NA; not available.

**Fig 1:**
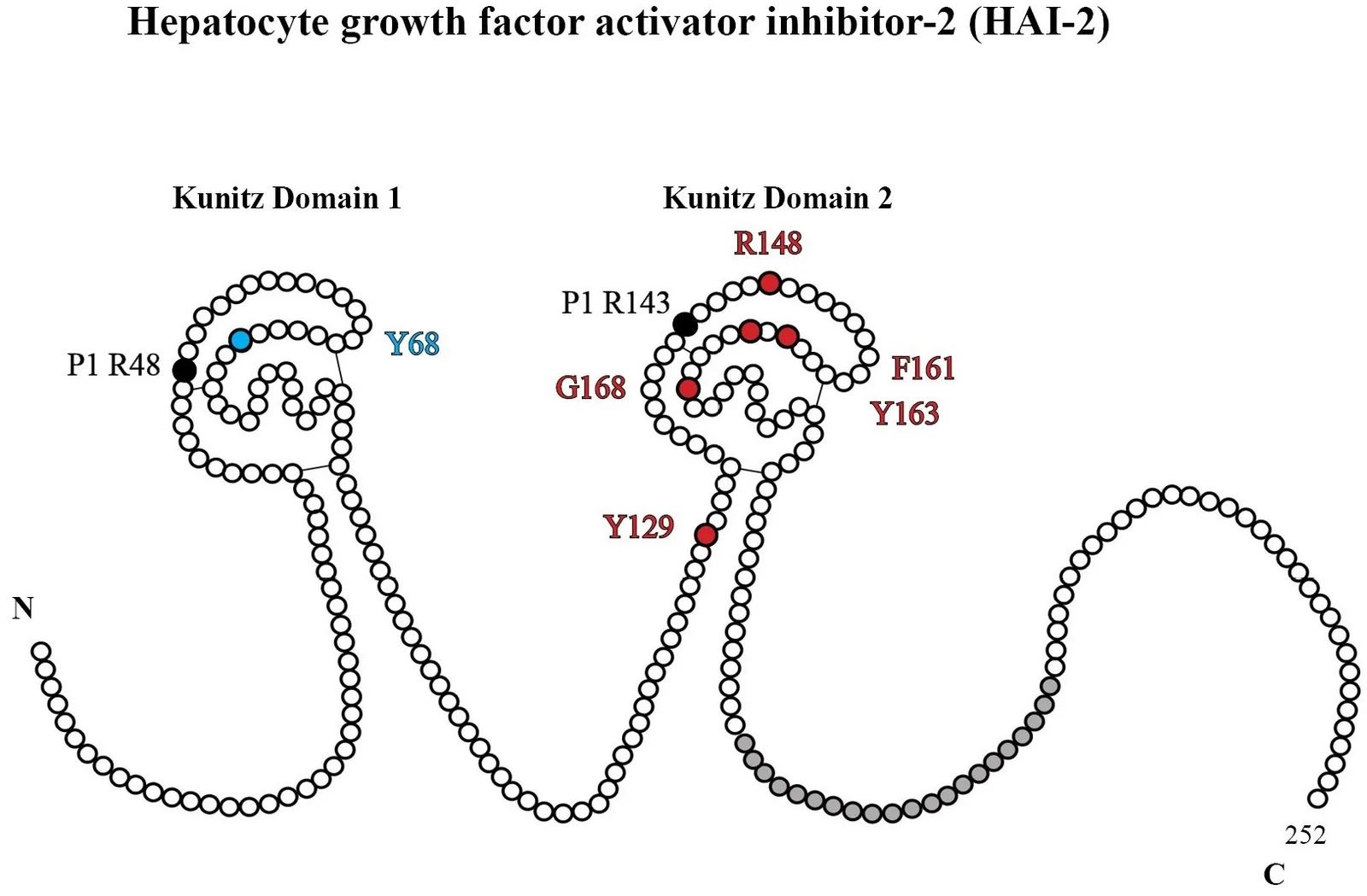
Schematic overview of the structure of human HAI-2. Each circle represents an amino acid residue. The transmembrane domain is shown as grey circles and the P1 residue of each KD is shown as a black circle. Amino acid residues altered by syndromic CSD-associated missense mutations are shown as red circles while the residue affected by the single non-syndromic CSD-associated missense mutation is shown as a blue circle. Black lines between amino acid residues indicate disulfide bonds.

HAI-2 is a type I transmembrane glycoprotein containing two extracellular Kunitz domains (KDs), annotated KD1 and KD2, respectively. KDs often display inhibitory activity against a range of serine proteases. A KD is composed of an approximately 60 amino acid residue polypeptide chain, which adopts a signature fold held together by three conserved disulfide bonds linking six cysteine residues in a C1-C6, C2-C4 and C3-C5 arrangement [16,17] (Fig 1). The protein structure of HAI-2 has not been experimentally determined but presumably acquires a fold like KDs in other proteins [18,19]. The two KDs are presumed to exert their inhibitory function independently of each other. We have previously shown that both KDs of HAI-2 inhibit the proteolytic activity of matriptase in a P1-dependent fashion but with different efficiency [20]. KDs contain a protease-binding loop, which consists of a stretch of amino acid residues (P3 to P3’) that mimics the substrate cleavage site of target serine proteases. Kunitz-type inhibitors insert the protease-binding loop of the KD into the active site of target proteases thus competitively inhibiting proteolytic cleavage of substrate [21]. In HAI-2, the P1 residues correspond to Arg48 and Arg143 in KD1 and KD2, respectively [22,23].

HAI-2 has been shown to inhibit several serine proteases including matriptase [24], prostasin [25], hepatocyte growth factor activator [23], tissue kallikrein [26] and matriptase-2 [27]. In a frog oocyte study, HAI-2 has been shown to inhibit nine different intestinal serine proteases: prostasin, matriptase, hepsin and transmembrane serine protease (Tmprss) 2, 3, 4, 11a, 13 and 15. Of these nine proteases, the CSD-causing HAI-2 variant Y163C had reduced inhibitory activity towards prostasin and Tmprss13 [28]. These findings suggest that CSD is caused by unregulated proteolytic activity resulting from the loss of HAI-2 inhibitory activity towards several proteases. In the present study, we have investigated the ability of HAI-2 variants to inhibit prostasin as a proxy for the group of proteases inhibited by wild-type HAI-2 but left uninhibited by CSD-causing HAI-2 variants. For a review on prostasin see [29].

Prostasin and matriptase are atypical serine proteases that are synthesized in a zymogen form that possesses low but measurable intrinsic proteolytic activity [20,25]. The proteolytically active zymogen form of both matriptase and prostasin can be inhibited by HAI-1 and HAI-2 [20,25]. Matriptase is synthesized in a non-SEA domain cleaved form. The SEA-domain of matriptase can undergo activation due to a proteolytic cleavage after Arg614 catalyzed by matriptase itself or by prostasin.

In addition to its role as a serine protease inhibitor, HAI-2 also possesses “ally protein function” towards matriptase [30,31], Tmprss13 [32] and hepsin [33]. The term “ally protein function” is elusive but describes the observation that a recombinantly expressed protease cannot be detected at the protein level in a cell line unless it is co-expressed with an ally protein, such as HAI-1 and HAI-2. Currently, HAI-1 and HAI-2 are the only identified ally proteins. In the present study, we will refer to ally protein function as a stabilization of matriptase by the ally protein although the actual mechanism is poorly understood.

Recently, a novel recessive HAI-2 variant, Y68C, has been reported to cause a non-syndromic form of congenital diarrhea and tufting enteropathy [15]. For the single known patient, the stool sodium level was not determined but the patient was shown to have hyponatremia [15] and will therefore be regarded as a case of non-syndromic CSD in the present study. Interestingly, this patient displayed a much milder phenotype compared to patients homozygous or compound heterozygous for HAI-2 variants causing syndromic CSD [2,7,15].

We have previously shown that three syndromic CSD HAI-2 variants are less efficient inhibitors of prostasin than wild-type HAI-2 [9]. This is also indicated by the lack of complex formation between prostasin and four different CSD-associated HAI-2 variants [34]. In the present study, we investigated the biochemical similarities and differences between the only known HAI-2 variant associated with non-syndromic CSD (Y68C) and four HAI-2 variants known to cause syndromic CSD (Y129C, R148H, F161V, and G168S). We found that all five HAI-2 variants causing CSD display reduced ability to inhibit prostasin regardless of whether they cause syndromic or non-syndromic CSD. In addition, the HAI-2 variant causing non-syndromic CSD (Y68C) also exhibits reduced ability to inhibit matriptase.

## Results

### CSD-associated HAI-2 variants and wild-type HAI-2 display a similar steady-state protein level and subcellular localization

The phenotypical differences between non-syndromic and syndromic CSD may be due to differences in the biochemical function of the associated HAI-2 variants. To investigate this, we first examined the steady-state protein level and the subcellular localization of the CSD-associated HAI-2 variants.

To test whether the non-syndromic CSD phenotype results from a reduced HAI-2 protein level in the cells, the steady-state protein level of the HAI-2 Y68C variant was assessed and compared to that of wild-type HAI-2 and HAI-2 variants (Y129C [14], R148H [13], F161V [9], and G168S) [7] (Fig 1). Two of the HAI-2 variants contain cysteine substitutions, and the presence of this extra cysteine residue may interfere with proper disulfide bond formation and folding [14]. Therefore, the HAI-2 Y68A and HAI-2 Y129A mutants were constructed and used as controls for the CSD-associated HAI-2 variants Y68C and Y129C.

**Fig 2:**
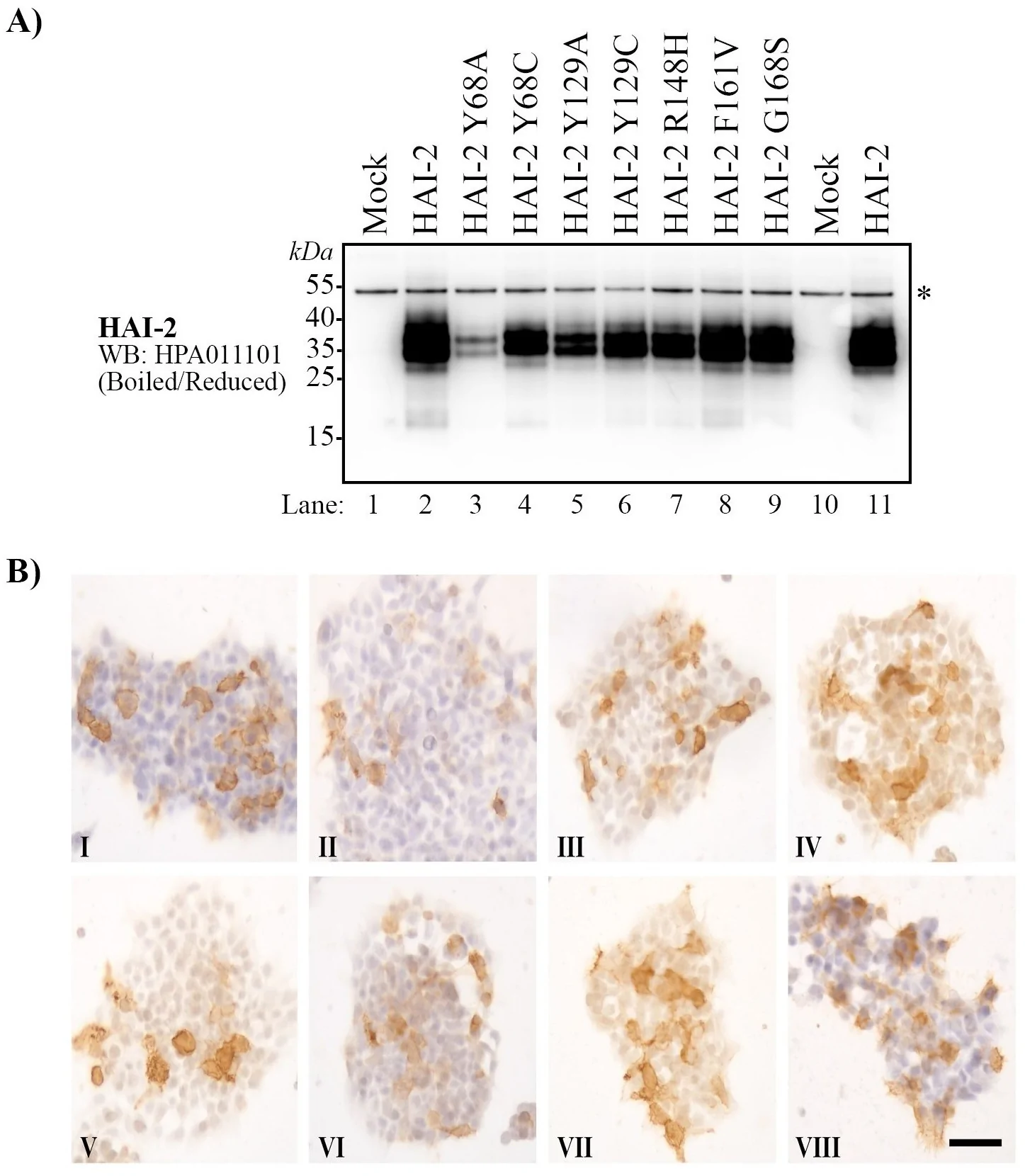
Similar steady-state protein level and subcellular localization of HAI-2 variants causing syndromic and non-syndromic CSD. A) HEK293 cells were transiently transfected with wild-type, variant or mutant HAI-2-encoding plasmids as indicated. Cell lysates were subjected to SDS-PAGE followed by western blotting using primary antibody against HAI-2. A protein band caused by unspecific binding is indicated by (*) to the right. A molecular mass marker is indicated to the left. Conclusions from these experiments are based on reproducible qualitative patterns across N = 3 experiments. B) Immunohistochemical staining of HAI-2 in HEK293 cells transiently transfected with wild-type, variant or mutant HAI-2-encoding plasmids; I) wild-type HAI-2, II) HAI-2 Y68A, III) HAI-2 Y68C, IV) HAI-2 Y129A, V) HAI-2 Y129C, VI) HAI-2 R148H, VII) HAI-2 F161V and VIII) HAI-2 G168S. Cells were counterstained with hematoxylin.

Plasmids encoding HAI-2 variants, mutants or wild-type HAI-2 were transiently transfected into HEK293 cells. 48 hours post transfection cells were either lysed and analyzed by western blotting (Fig 2A) or fixed and immunohistochemically stained (Fig 2B). In both cases, primary antibodies against HAI-2 were used.

The results showed that the non-syndromic CSD HAI-2 variant (Y68C), all the syndromic CSD HAI-2 variants (Y129C, R148H, F161V and G168S), and the control HAI-2 Y129A mutant could be detected by western blotting and exhibited a steady-state protein level similar to that of wild-type HAI-2 (Fig 2A compare lane 2 and 11 to lanes 4−9). In contrast, the control HAI-2 mutant, Y68A, was detected at a lower steady-state level than the variants and wild-type HAI-2 (Fig 2A lane 3). The conclusions from this experiment are based on reproducible qualitative patterns across N = 3 experiments. These findings indicated that changes in the steady-state protein level of HAI-2 variants cannot explain the phenotypical differences between the HAI-2 Y68C variant, the variants, Y129C, R148H, F161V and G168S and wild-type HAI-2. Only the control HAI-2 Y68A mutant, which has not been detected in CSD patients, displays a lower steady-state protein level.

It is possible that the phenotypical differences between non-syndromic and syndromic CSD could be due to differences in the subcellular localization of associated HAI-2 variants. We have previously shown that wild-type HAI-2 is located mainly in the ER and, to a minor degree, on the apical plasma membrane of polarized epithelial cells [35]. A similar distribution between ER and plasma membrane was observed when CSD-associated HAI-2 variants were examined in several non-polarized cell lines [36]. As mutations may introduce difficulties for the protein to fold correctly resulting in lack of transport competencies down the secretor pathway, immunocytochemistry was performed to ensure that mutants display the HAI-2 immunoreactivity as a thin staining along the cell periphery, as an indicator that proper folding has been obtained. in addition to the intracellular staining, as seen for the wild-type protein.

Immunohistochemical staining of transiently transfected HEK293 cells showed that of all CSD-associated HAI-2 variants display a weak staining along the perimeter of the cells in addition to intracellular staining similar to the staining of cells expressing wild-type HAI-2 (Fig 2B).

In the secretory pathway misfolded proteins primarily localize to the ER, which is also where the majority of wild-type HAI-2 is localized [35,36]. This subcellular distribution of HAI-2 is due to the presence of two ER retention signals and an incomplete plasma membrane exportation motif in the cytoplasmic tail of HAI-2 [36]. However, the weak pericellular staining could be detected for both the non-syndromic and syndromic CSD HAI-2 variants and wild-type HAI-2. These findings suggest that the phenotypical differences between non-syndromic and syndromic CSD are not caused by altered subcellular localization of the associated HAI-2 variants.

### All CSD-causing HAI-2 variants exhibit reduced ability to inhibit prostasin

We investigated whether the non-syndromic HAI-2 variant Y68C and the four syndromic CSD HAI-2 variants exhibit decreased inhibitory capacity towards prostasin using a previously described assay [9] with a slight modification. The assay uses the catalytically inactive matriptase variant S805A as substrate. Wild-type matriptase is substrate for prostasin and for itself, as it is able to trans auto-activate. Thus, a specific prostasin substrate can be created by mutating the active site serine in wild-type matriptase, generating matriptase S805A, whereby trans auto-activation is prevented. However, matriptase S805A is only specific for prostasin in cells not expressing wild-type matriptase, such as HEK293 cells [25]. Prostasin catalyzed cleavage of matriptase S805A generates the 30 kDa SPD fragment of matriptase. A proteolytically active zymogen-locked prostasin variant (prostasin R44Q) was used instead of wild-type prostasin previously used in this assay [9]. In both cases, zymogen prostasin functions as the proteolytic active enzyme catalyzing the cleavage of matriptase S805A as wild-type prostasin is not activated under these circumstances [25].

**Fig 3:**
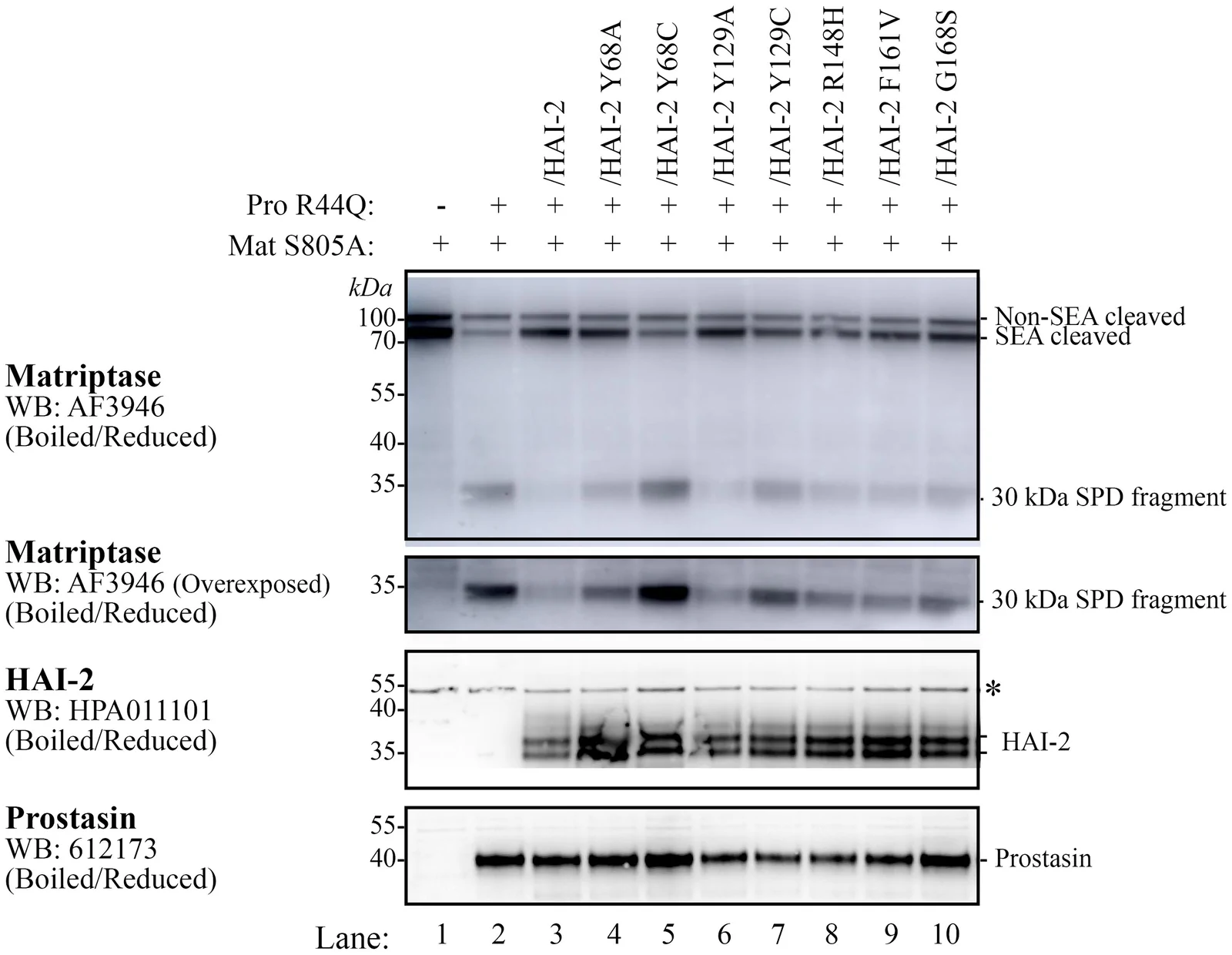
CSD-causing HAI-2 variants display a reduced ability to inhibit prostasin. HEK293 cells were transiently transfected with plasmids encoding matriptase S805A (Mat S805A), zymogen-locked prostasin (Pro R44Q) and wild-type or CSD-associated HAI-2 variants or HAI-2 mutants in the combinations indicated above. Cell lysates were analyzed by SDS-PAGE and western blotting using primary antibodies as indicated. Matriptase can be detected in three distinct forms: full-length (non-SEA domain-cleaved), SEA domain-cleaved matriptase and the 30 kDa matriptase fragment generated by prostasin-catalyzed cleavage roughly equivalent to the serine protease domain (SPD). An overexposed version of the matriptase membrane at the position around the 30 kDa matriptase band is included (Matriptase Overexposed). An unspecific band is indicated by (*). The conclusions from this figure are based on reproducible qualitative patterns across N = 3 independent experiments. Molecular mass markers are shown to the left and the positions of the three forms of matriptase as well as prostasin and HAI-2 are indicated to the right.

HEK293 cells were transiently transfected with matriptase S805A, prostasin R44Q and either a HAI-2 variant, mutant or wild-type HAI-2. Cells were lysed 48 hours post transfection, and cell lysates were analyzed by western blotting (Fig 3).

When expressed alone, matriptase S805A was detected as two high molecular bands corresponding to the full-length (non-SEA domain-cleaved) and the SEA domain-cleaved form of matriptase (Fig 3 lane 1). However, no 30 kDa SPD fragment could be detected showing that no 30 kDa SPD fragment is generated by endogenous expressed proteases. In addition, cells transfected with matriptase S805A display no prostasin or HAI-2 immunoreactivity indicating no or low endogenous expression of these two proteins (Fig 3 lane 1). Co-expression of matriptase S805A with prostasin R44Q (zymogen-locked prostasin) resulted in the generation of an additional matriptase-immunoreactive band, the 30 kDa SPD fragment (Fig 3 lane 2). In contrast, no 30 kDa matriptase fragment could be detected when matriptase S805A and prostasin R44Q were co-expressed with wild-type HAI-2 (Fig 3, lane 3). These findings confirmed that wild-type HAI-2 efficiently inhibits the proteolytic activity of zymogen prostasin. The ability of HAI-2 variants was investigated in a similar manner (Fig 3, lanes 4−10. Both the non-syndromic CSD HAI-2 variant (Y68C) and the syndromic CSD HAI-2 variants (Y129C, R148H, F161V and G168S) displayed a reduced ability to inhibit prostasin compared to wild-type HAI-2 (Fig 3 compare lane 3 to lanes 5, 7−10 when overexposed). The control HAI-2 mutant Y68A and Y129A also exhibited a reduced ability to inhibit prostasin (Fig 3 compare lane 3 to lanes 4 and 6 when overexposed). Both control mutants, Y68A and Y129A, were more efficient prostasin inhibitors than their corresponding CSD-associated HAI-2 variants Y68C and Y129C, respectively (Fig 3 lanes 4 and 6 versus lanes 5 and 7). The conclusions from this experiment are based on reproducible qualitative patterns across N = 3 experiments.

In conclusion, all CSD-associated HAI-2 variants investigated, regardless of whether they cause syndromic or non-syndromic CSD, displayed a reduced inhibitory activity towards prostasin. These findings suggest that reduced inhibitory ability towards prostasin may be a reliable proxy for the detection of CSD-causing HAI-2 variants.

### The non-syndromic CSD HAI-2 Y68C variant is unable to inhibit matriptase

It was investigated whether the newly discovered non-syndromic and syndromic CSD HAI-2 variants were capable of inhibiting matriptase. All CSD-associated HAI-2 variants investigated so far have been shown to inhibit matriptase with an efficiency similar to wild-type HAI-2 [9].

**Fig 4:**
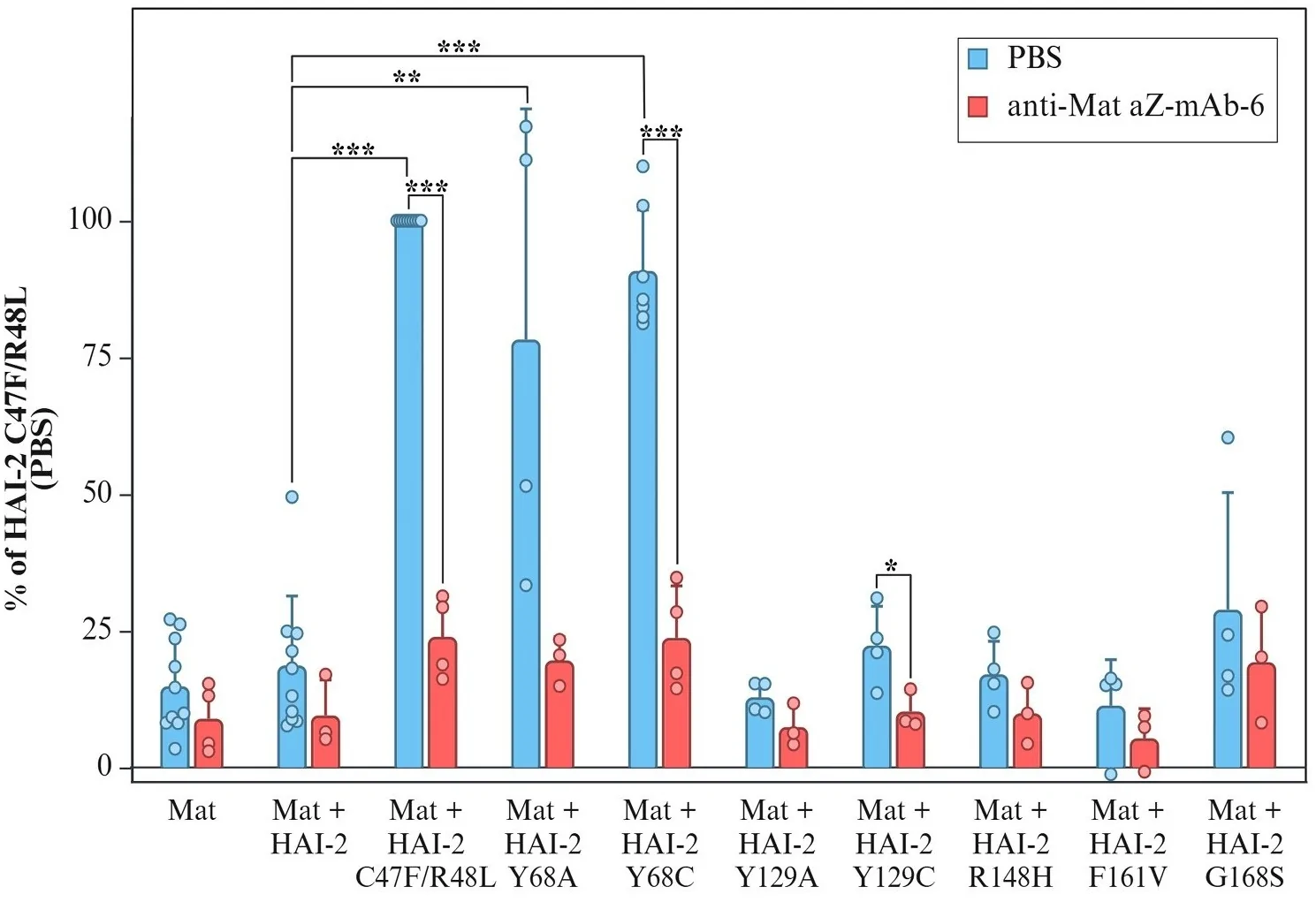
The non-syndromic CSD HAI-2 Y68C variant exhibits a highly reduced ability to inhibit matriptase. HEK293 cells were transiently transfected with wild-type matriptase alone or in combination with wild-type HAI-2 or CSD associated HAI-2 variants or mutants as indicated above. Cell lysates were incubated with either PBS or the matriptase antibody aZ-mAb-6, which inhibits matriptase proteolytical activity, before the addition of the general serine protease chromogenic substrate, S-2288. The peptidolytic activity of cell lysates was detected as a change in absorbance at 405 nm resulting from proteolysis of the chromogenic substrate. Each column indicates the mean + /- SD of the peptidolytic activity measured in mAU/min in the absence (blue columns) or presence (red columns) of the aZ-mAb-6 antibody. Differences in the rate of peptidolytic activity were analysed using un-paired t-test (* p < 0.05, ** p < 0.01, *** p < 0.001) without correction for multiple comparisons. N for each column -/ + antibody is shown in brackets (Mat (10/4), Mat + HAI-2 (10/3), Mat + HAI-2 C47F/R48L (10/4), Mat + HAI-2 Y68A (4/3), Mat + HAI-2 Y68C (7/3), Mat + HAI-2 Y129A (4/3), Mat + HAI-2 Y129C (4/3), Mat + HAI-2 R148H (4/3), Mat + HAI-2 F161V (4/3) and Mat + HAI-2 G168S (4/3). The figure displays 10 independent experiments, all of which included control and a subset of the samples. The figure was created with BioRender.com.

In this assay, the matriptase activity of cell lysates was determined as cleavage rate of a non-specific serine protease peptide substrate in the presence or absence of the aZ-mAb-6 antibody, which specifically inhibits both activated and zymogen matriptase [37]. HEK293 cells were transiently transfected with plasmids encoding matriptase alone or in combination with plasmids encoding either wild-type or CSD-associated variants or mutants of HAI-2. The HEK293 cells were lysed 48 hours post transfection, and the cell lysates were subsequently incubated with either aZ-mAb-6 antibody or PBS (vehicle) before measuring the peptidolytic activity. The mean cleavage rate and standard deviation were calculated for the measurements of each transfection condition in the presence or absence of aZ-mAb-6 antibody (Fig 4).

Three types of controls were included: cells transfected with matriptase alone, cells transfected with matriptase and wild-type HAI-2, and cells transfected with matriptase and HAI-2 C47F/R48L. These three controls were included to evaluate the background and range of the assay. It has previously been shown that matriptase is hardly detectable in lysates of HEK293 cells transfected with an expression plasmid for matriptase alone using western blot [9] which is caused by the need of an ally protein like HAI-2 to stabilize the matriptase protein [30]. However, theoretically a minute amounts of matriptase present may activate other serine proteases and thus affect the background level or give raise to matriptase specific activity in low amounts. Analysis of the lysate of cells expressing only matriptase showed no difference between the lysates analyzed in the presence and absence of the inhibitory antibody aZ-mAb-6, thus no matriptase specific activity could be detected, which is expected as there are no ally protein present to stabilize the matriptase protein. However, peptidolytic activity that could not be inhibited by the aZ-mAb-6 antibody was detected and indicates the background of the assay, which most likely stems from endogenously expressed serine proteases recognizing the general serine protease substrate used. Extracts from cells co-expressing matriptase and wild-type HAI-2 displayed similar low peptidolytic activity, which was unaffected by the aZ-mAb-6 antibody as also shown previously [37]. This shows that no matriptase activity could be detected in the lysates and that other proteases, endogenously expressed in HEK293 cells and not inhibited by the matriptase-specific aZ-mAb-6 antibody generates a background of the assay as seen previously [9,37]. It was also previously shown that HAI-2 is able to provide ally protein support to matriptase under these circumstances as it can be detected by western blot.

In contrast, extracts from cells co-expressing matriptase and HAI-2 C47F/R48L exhibited a high peptidolytic activity in the absence of aZ-mAb-6 antibody. Comparing the peptidolytic activity in the presence and absence of aZ-mAb-6 antibody showed a statistically significant decrease in peptidolytic activity upon antibody treatment (p < 0.001) (Fig 4). Furthermore, a statistically significant difference in peptidolytic activity was detected between the lysate of cells co-expressing matriptase and wild-type HAI-2 when compared to cells expressing matriptase and HAI-2 C47F/R48L in the absence of aZ-mAb-6 antibody treatment (p < 0.001) (Fig 4). This shows the range of the assay and that HAI-2 C47F/R48L has a reduced inhibitory activity towards matriptase but displays ally protein function causing stabilization of the matriptase protein. Wild-type HAI-2 and HAI-2 C47F/R48L was included here as a positive and negative control for inhibition of matriptase activity as they both previously has been shown to display ally protein function towards matriptase [9]. Thus HAI-2 not only stabilizes the matriptase protein, but it may also inhibit the proteolytic activity of matriptase as shown for wild-type HAI-2 but not for HAI-2 C47F/R48L.

The extracts from cells co-expressing matriptase and the non-syndromic CSD HAI-2 Y68C variant also displayed high peptidolytic activity in the absence of aZ-mAb-6 antibody. A statistically significant decrease in peptidolytic activity could be detected when comparing the lysate of cells co-expressing Matriptase and HAI-2 Y68C incubated without or with aZ-mAb-6 antibody (p < 0.001) (Fig 4). Moreover, in the absence of aZ-mAb-6 antibody, a statistically significant difference in peptidolytic activity was found between the lysate of cells co-expressing matriptase and wild-type HAI-2 as compared to cells co-expressing matriptase and HAI-2 Y68C (p < 0.001) (Fig 4). These results show that the non-syndromic CSD HAI-2 Y68C variant displays reduced ability to inhibit matriptase.

In addition, a statistically significant increase in peptidolytic activity was seen when comparing the lysate of cells co-expressing matriptase and wild-type HAI-2 with cells expressing matriptase and the control, HAI-2 Y68A mutant in the absence of aZ-mAb-6 antibody (p < 0.01) (Fig 4).

The extracts from cells co-expressing matriptase and either the control HAI-2 Y129A mutant or one of the syndromic CSD HAI-2 variants (R148H, F161V and G168S) exhibited low peptidolytic activity, which was unaffected by the presence of the aZ-mAb-6 antibody (Fig 4). A statistically significant decrease in peptidolytic activity was detected in the extracts of cells co-expressing matriptase and the HAI-2 Y129C variant when comparing the activity detected in the absence and presence of aZ-mAb-6 antibody (p < 0.05) (Fig 4).

In conclusion, the non-syndromic CSD HAI-2 variant (Y68C) and possibly also to a minor extent the syndromic CSD HAI-2 Y129C variant exhibited a reduced ability to inhibit matriptase. Whereas the syndromic CSD HAI-2 variants (R148H, F161V and G168S) were all capable of inhibiting matriptase as efficiently as wild-type HAI-2.

### HAI-2 inhibits prostasin using an inhibitory mechanism unlike other Kunitz type inhibitors

To determine which of the KDs in HAI-2 are inhibitory active towards prostasin, mutations in the P1 amino acid residues in KD1 and/or KD2 were studied in addition to the P2 variant in KD1, C47F. HAI-2 C47F is a naturally occurring human HAI-2 variant with an unknown phenotype [38]. We have previously shown that HAI-2 C47F/R48L displays reduced ability to inhibit prostasin [9]. The prostasin inhibitory capacity was determined as described above for Fig 3.

**Fig 5:**
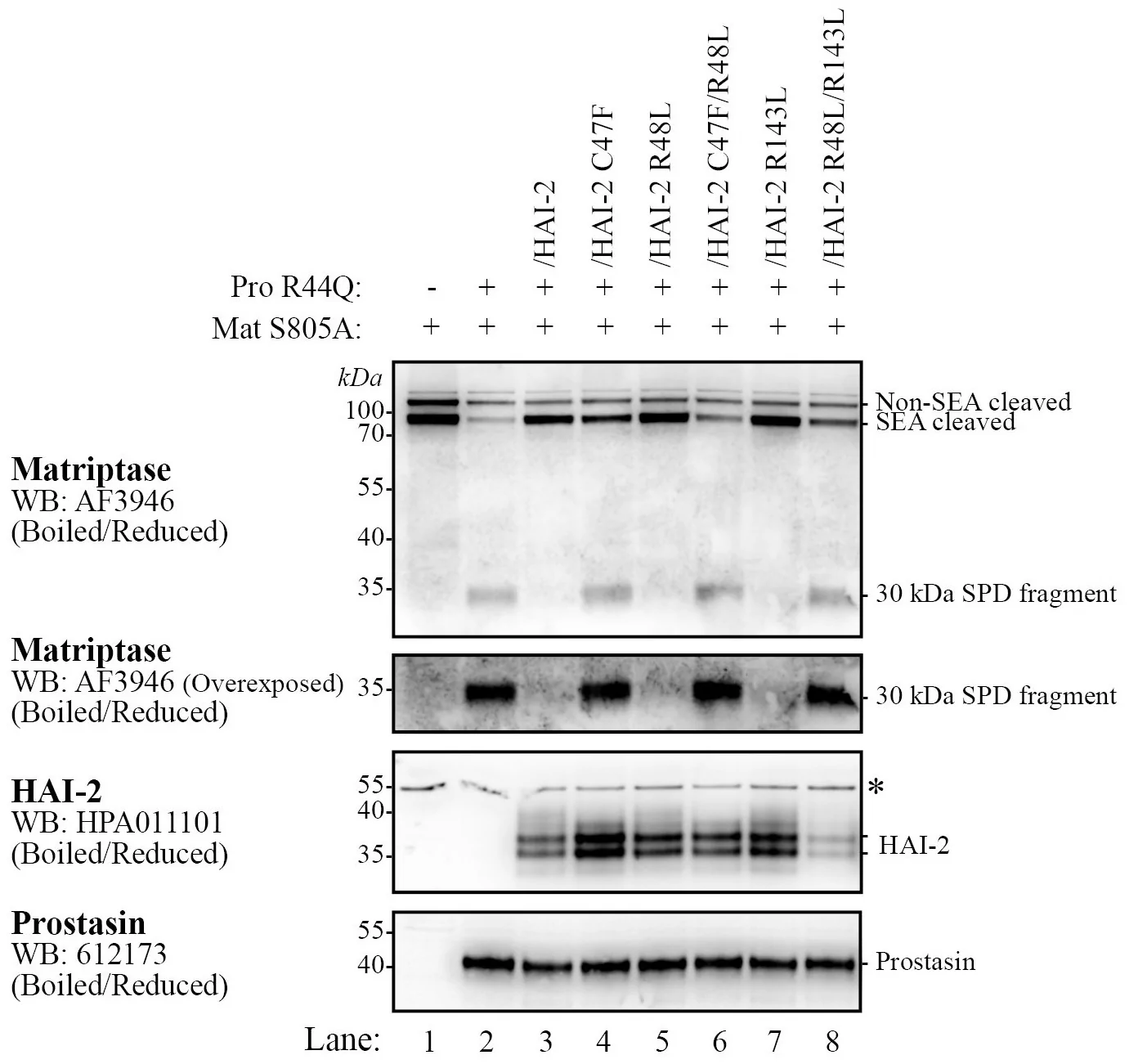
HAI-2 inhibits prostasin in an unconventional way. HEK293 cells were transiently transfected with plasmids encoding matriptase S805A (Mat S805A), zymogen-locked prostasin (Pro R44Q) and wild-type or P1/P2 HAI-2 mutants or variants. Cell lysates were subjected to SDS-PAGE and western blotting using primary antibodies as indicated. An overexposed version of the matriptase membrane is included to ease the detection of the 30 kDa matriptase band (Matriptase overexposed). A protein band caused by unspecific binding is indicated by (*) to the right. Molecular mass markers are shown to the left and the positions of matriptase, prostasin and HAI-2 are indicated to the right. The conclusions from this figure are based on reproducible qualitative patterns across N = 3 independent experiments.

The results showed that HAI-2 mutants harboring a single mutation in the P1 residue of either KD1 (HAI-2 R48L) or KD2 (HAI-2 R143L) were capable of inhibiting zymogen prostasin with an efficiency similar to the wild-type HAI-2 (Fig 5 compare lane 3 to lane 5 and 7). A HAI-2 mutant with both P1 residues mutated (HAI-2 R48L/R143L) was present at a much lower steady-state level than wild-type HAI-2 and was unable to inhibit prostasin (Fig 5 compare lane 3 to lane 8). Abolishment of a conserved disulfide bond in HAI-2 by the C47F variant either alone or in combination with the R48L mutation (HAI-2 C47F/R48L) also caused reduced inhibitory activity towards prostasin (Fig 5 compare lane 3 to lane 4 and 6).

For protease inhibitors like HAI-2, the two inhibitory domains are presumed to function independently of each other, with the P1 residue functioning as an important determinant of specificity. Theoretically, if both KDs are capable of inhibiting a particular protease, a mutation in one domain should not impair overall inhibition, as the remaining functional KD is expected to compensate. In accordance with this the finding that HAI-2 mutated in the P1 amino acid residue in either individual KD retains full inhibitory capacity suggests that both KD1 and KD2 of HAI-2 are inhibitory active against prostasin and that one functional KD can effectively substitute for a mutated one. However, HAI-2 mutated in both P1 residues (HAI-2 R48L/R143L) were unable to inhibit prostasin. This HAI-2 mutant is not expressed at the same level as wild-type HAI-2. Thus, it is uncertain whether the reduced prostasin inhibition is due to the mutations of the P1 residues or the low steady-state level of this HAI-2 mutant.

However, there are several observations that are difficult to reconcile with the model of two functionally independent KDs. For instance, HAI-2 carrying one of several mutations (Y129A, Y129 C, R148H, F161V or G168S) located in the linker between the two domains or in KD2 displays reduced inhibitory activity towards prostasin despite the presence of a wild-type KD1, which was shown to be fully functional as it inhibits matriptase (this study and [9]). Furthermore, HAI-2 carrying mutations in KD1 (C47F, C47F/R48L,Y68A or Y68C) displays reduced inhibitory ability towards prostasin despite the presence of a wild-type KD2. Many of the mutations in HAI-2, which cause a reduced ability to inhibit prostasin, affect amino acid residues that are involved in forming disulphide bonds or intradomain interactions, which hold the KD together (Table 2). Altering these amino acid residues would loosen the structure of the KDs indicating that preserving the domain structure of both KDs is essential for prostasin inhibition by HAI-2.

**Table 2:** Amino acid interactions and inhibitory function of CSD-associated HAI-2 variants.

HAI-2 variant	Abbreviation	Protein domain	Amino acid interactions in WT HAI-2	Reduced inhibition of prostasin	Reduced inhibition of matriptase
p.Tyr68Cys	Y68C	KD1	Met51, Gly73	Yes	Yes
p.Tyr129Cys	Y129C	Linker /KD2 *	Phe127, Tyr132, Cys133, Thr134	Yes	To a minor degree
p.Arg148His	R148H	KD2	Phe161, Ser172	Yes	No
p.Phe161Val	F161V	KD2	Arg148	Yes	No
p.Tyr163Cys	Y163C	KD2	Phe146, Arg167	Yes	No
p.Gly168Ser	G168S	KD2	None	Yes	No

* According to AlphaFold this variant is localized in the linker region between KD1 and KD2. However, it interacts with amino acid residues in KD2 suggesting that it is a part of this domain.

Collectively, these findings suggests that HAI-2 inhibits prostasin in a manner that involves both KDs and is different from that of classical Kunitz type inhibitors and the way HAI-2 inhibits matriptase [20]. One possible explanation is that the binding interface between HAI-2 and protease may vary depending on the target protease. In the case of prostasin, this interface could potentially span both KDs.

## Discussion

### HAI-2 Y68C likely allows matriptase-catalyzed activation of prostasin in the early secretory pathway

**Fig 6:**
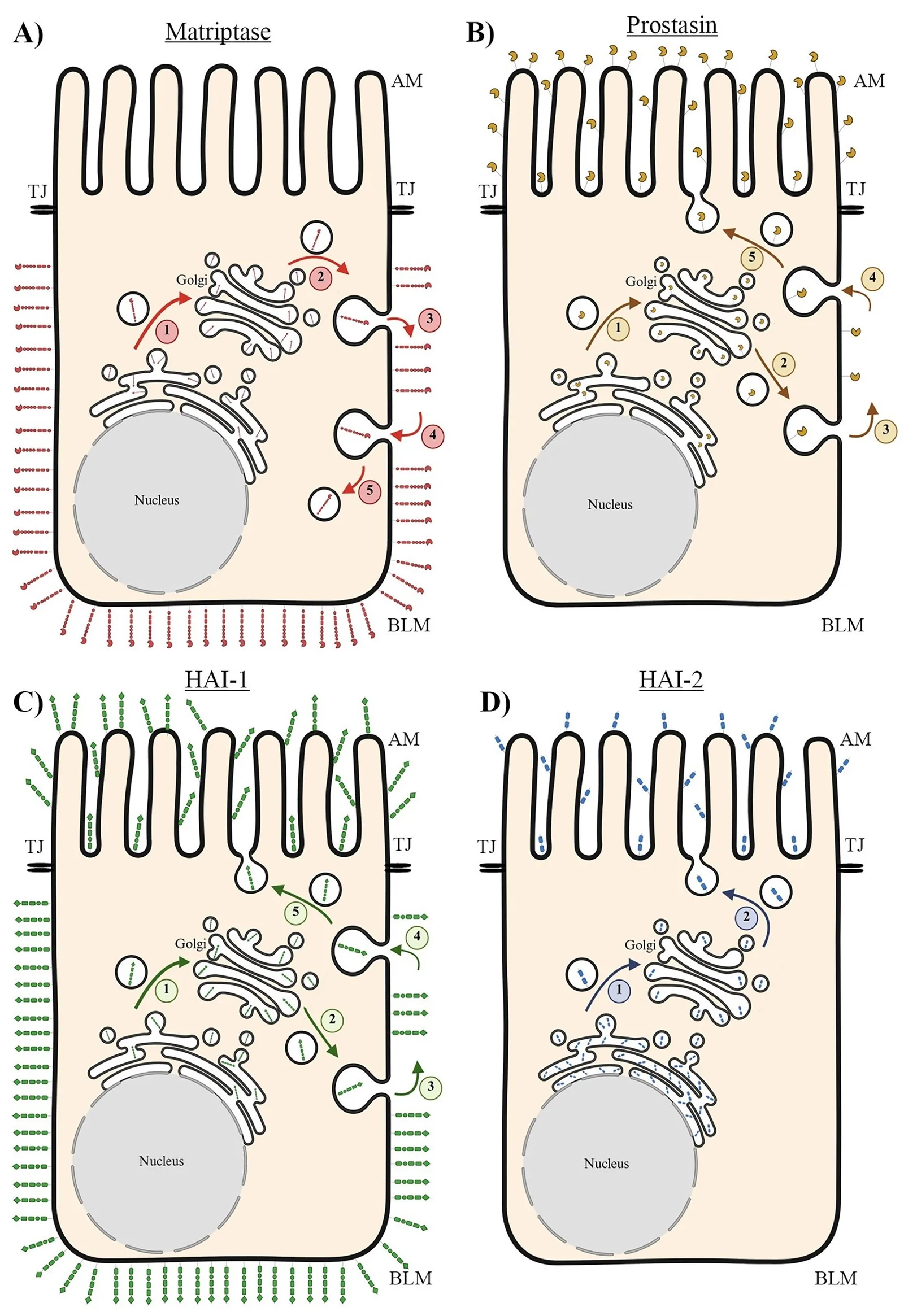
Schematic overview of the intracellular transport pathways and distribution between the apical and basolateral plasma membrane at steady state for matriptase, prostasin, HAI-1 and HAI-2. A) Matriptase is synthesized on the rER (1) before being transported through the secretory pathway (2) to the basolateral plasma membrane (3). Some matriptase molecules are subsequently endocytosed (4) and transported to an unidentified vesicular compartment (5). B) and C) Prostasin and HAI-1 are both synthesized on the rER (1) and transported through the secretory pathway (2) to the basolateral plasma membrane (3). From where prostasin and HAI-1 are endocytosed (4) and transcytosed (5) to the apical plasma membrane. D) HAI-2 is synthesized on the rER (1), where the majority remains, but a minor fraction of HAI-2 is transported though the secretory pathway to the Golgi apparatus and subsequently to the apical plasma membrane (2). Abbreviations: AM = apical plasma membrane, BLM = basolateral plasma membrane, TJ = tight junctions. The figure was created with BioRender.com.

Matriptase, prostasin and both of their common inhibitors, HAI-1 and HAI- 2, are expressed in enterocytes, where their intracellular transport pathways have been mapped as depicted (Fig 6) [35,39,40]. The arrows indicate the transport pathway and the coloured icons indicate the distribution between the apical and the basolateral plasma membrane at steady state. Zymogen matriptase, zymogen prostasin, HAI-1 and HAI-2 are all membrane bound and synthesized on the rER and it is thus possible for them to interact in the ER lumen and in the early secretory pathway. This seems to take place as complexes between either HAI-1 or HAI-2 with either the proteolytically active zymogen prostasin [25] or the proteolytically active zymogen matriptase [20] can be detected in cell extracts. There are two transport pathways to the apical plasma membrane in polarized epithelial cells: a direct pathway from the trans-Golgi network to the apical plasma membrane, used by HAI-2 [35] and the transcytotic pathway, which leads from the trans-Golgi network to the basolateral plasma membrane followed by endocytosis and transcytosis to the apical plasma membrane, used by prostasin [39] and HAI-1 [40]. Matriptase is also transported to the basolateral plasma membrane and endocytosed but not transcytosed [39].

Presuming that the biochemical difference between the variant causing non-syndromic CSD and the ones causing syndromic CSD described in this study are causing the difference in phenotype, how can this be explained? In contrast to the other variants, HAI-2 Y68C causes matriptase to be left un-inhibited. The ER lumen and the early secretory pathway are the only subcellular locations shared by HAI-2 and matriptase (Fig 6). We have previously shown that matriptase is able to cleave and activate prostasin [25]. We thus hypothesize that in individuals homozygous for the HAI-2 Y68C variant, some level of un-inhibited matriptase is present in the early secretory pathway despite the presence of HAI-1, which is also able to inhibit matriptase [20]. Matriptase-catalyzed activation of prostasin is thus likely to take place in the early secretory pathway. It is at present unclear exactly how this may lead to a change in the phenotype for carriers of this HAI-2 variant.

Proteases with active zymogen forms are rare and understudied, but as far as we know, the active zymogen and the activated form both exhibit catalytic activity with similar substrate specificities [20,25], yet differ in catalytic activity [37]. However. it has been clearly shown that different phenotypes can be observed in mice expressing only zymogen locked prostasin as compared to mice expressing wild-type prostasin, which can be activated [41]. This clearly shows that activation of prostasin affects the phenotype. Similar results have been obtained for matriptase [42]. It is thus possible that the difference between the syndromic and the non syndromic CSD associated with HAI-2 variants is caused by the increased matriptase catalysed activation of prostasin.

### HAI-2 variants cause CSD due to un-opposed proteolytic activity

Throughout this study HEK293 cells were used for the assays detecting prostasin and matriptase activity since this is the only cell line where these assays are known to work. However, the processes we analyze take place in intestinal cells, where the assays used cannot be expected to work due to endogenous expression of matriptase. The conclusions drawn here are thus based on the presumption that the processes studies behave the same in HEK293 cells and in intestinal cells. Also, this analysis is based on a single assay for matriptase and a single assay for prostasin. However, our data show that all 6 CSD-causing HAI-2 variants investigated in this study exhibit a reduced ability to inhibit prostasin. The effects of these CSD-causing HAI-2 variants on prostasin likely extend to a subset of serine proteases, as evidenced by the Y163C variant, which loses inhibitory activity against two out of nine intestinal proteases in a Xenopus oocyte expression system [28]. Evidence suggests that Tmprss13, like prostasin and matriptase, is produced in an active zymogen form [32] and is thus proteolytically active before activation has taken place. As a result, HAI-2 co-localizes with the active zymogen form of target proteases like prostasin, matriptase and Tmprss13 both in the ER-lumen, the early secretory pathway and, for prostasin and Tmprss13, also on the apical plasma membrane (Fig 6). We hypothesize that individuals suffering from CSD, the target proteases in those locations are less inhibited which allows un-opposed proteolytical activity to proteolytically modify or degrade proteins in their vicinity. This contrasts with individuals homozygous for the wild-type HAI-2, who are less prone to un-opposed proteolytic enzymes.

### Potential treatment of CSD caused by variants of the *SPINT*2 gene

CSD is tightly connected to the ability of molecules in the apical plasma membrane of enterocytes to facilitate the uptake of sodium from the gut lumen. This is directly demonstrated by variants of the genes encoding NHE3 and guanylate cyclase C, both resulting in reduced activity of the sodium-hydrogen exchanger, NHE3, whereby sodium uptake is reduced. Also, individuals homozygous for any one of the 6 HAI-2 variants causing CSD exhibit reduced uptake of sodium [1].

**Fig 7:**
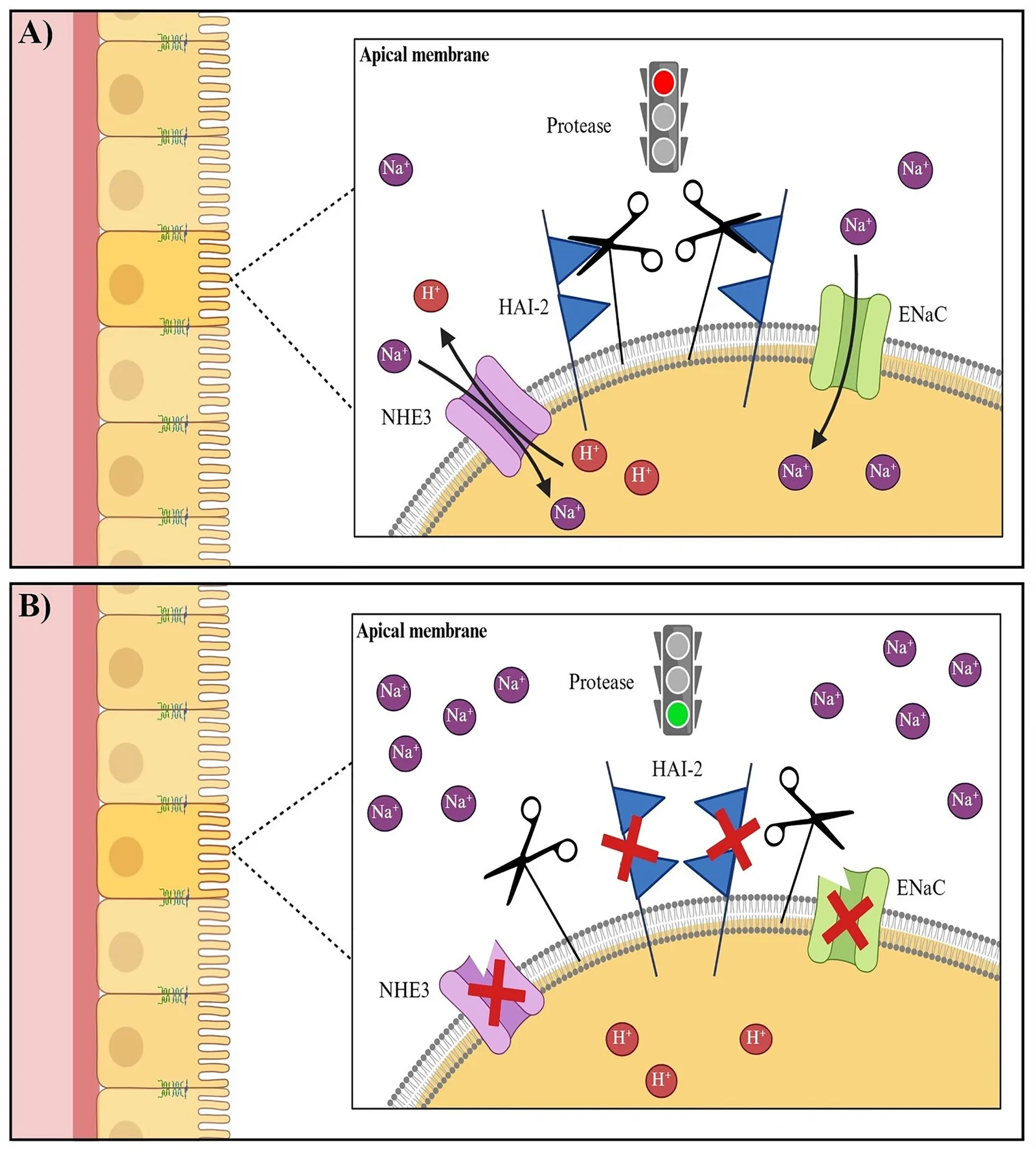
Hypothesis for the involvement of HAI-2 in CSD. A) Under normal conditions, HAI-2 inhibits prostasin and other similar proteases (symbolized by a scissor) on the apical plasma membrane of enterocytes keeping proteolysis low (indicated by the red traffic light). This inhibition prevents the degradation of NHE3/ENaC and/or other proteins thus promoting efficient uptake of sodium from the gut lumen. B) In the case of CSD, the HAI-2 variants exhibit a compromised inhibitory capacity leaving prostasin and similar proteases un-inhibited, resulting in higher proteolytic activity (indicated by the green traffic light). We hypothesize that this un-opposed proteolytic activity leads to the degradation of proteins involved in intestinal sodium uptake such as NHE3 and/or ENaC, resulting in decreased sodium absorption and development of sodium diarrhea. The figure was created with BioRender.com.

HAI-2 located in the early secretory pathway and on the apical plasma membrane of enterocytes (Fig 6) have their inhibitory KDs exposed to the lumen of the organelle or the gut lumen [35]. Consequently, certain proteases, including at least prostasin, in the secretory pathway and on the apical plasma membrane of enterocytes are less efficiently inhibited when co-expressed with a CSD causing HAI-2 variant as compared to in the presence of wild-type HAI-2. We hypothesize that the presence of CSD causing variants results in an increased un-inhibited proteolytic activity that inactivates NHE3, ENaC and/or other proteins involved in the uptake of sodium ions from the gut lumen (Fig 7). It seems like the same mechanism works for both syndromic and non-syndromic CSD as all CSD causing HAI-2 variants studied exhibit reduced inhibitory activity towards prostasin (this study and [9]) which in this context likely represents a group of proteases, possibly also including Tmprss13.

How can we help patients with CSD? We suggest investigating whether individuals with CSD can be treated by the oral intake of a protease inhibitor to mimic the wild-type HAI-2 inhibitory activity. The protease inhibitor may be in the form of a peptide inhibitor or a protein inhibitor. Both types of inhibitors often have broad specificity when targeting serine proteases, which are known for their overlapping substrate preferences. This broad specificity can be advantageous in this context, since CSD is most likely caused by the lack of inhibition of several proteases located on the apical plasma membrane. Such treatment is not likely to fully cure CSD as the un-opposed proteases are also co-located with sodium-transporters in the early secretory pathway but may help limit the extracellular proteolysis of proteins involved in the uptake of sodium from the gut lumen.

## Materials and methods

### Cell culturing, transfections and DNA constructs

The human embryonic kidney cell line HEK293 was cultured in Dulbecco’s Modified Eagle’s Medium (DMEM) 1965 supplemented with 1% L-glutamine, 10% fetal bovine serum and 1% penicillin/streptomycin at 37 °C in an atmosphere of 5% CO_2_. For experiments, cells grew to 80% confluence. One day prior to transfection, 500.000–600.000 cells per well were seeded into 6-well Nunclon^TM^ Cell-Culture Treated Multidishes (cat. no. 140675). HEK293 cells were transiently transfected using either Lipofectamine^TM^ 2000 (Invitrogen) or Lipofectamine 3000^TM^ (Invitrogen) in accordance with the recommendations of the manufacturer. In the case of co-transfections, the same amount of each individual plasmid was applied, and empty mock plasmids were added to ensure that the same total amount of plasmid was used for each transfection. Full-length human matriptase and HAI-2 cDNAs were incorporated into pCDNA3.1 plasmid vectors while cDNA encoding full-length human prostasin was incorporated into pIRES-eGFP plasmid vectors. The HAI-2 cDNA used in this study includes a naturally occurring SNP, rs11548457, which generates a V200L amino acid substitution in the protein product. For mock transfections, empty pCDNA3.1 plasmid vectors were used. The Y68A, Y68C, Y129A, Y129C, R148H, F161V, Y163C and G168S mutations were introduced into the HAI-2 encoding cDNA by site-directed mutagenesis using either the GeneArt^®^ Site-Directed Mutagenesis System (cat.no. A13282, Life Technologies) or the GeneArt^®^ Site-Directed Mutagenesis PLUS Kit (cat.no. A14604, Life Technologies). Both mutagenesis kits were used in accordance with the protocols supplied by the manufacturer. The obtained mutations were verified by sequencing. The remaining matriptase, prostasin and HAI-2 variants used in this study have all been described in other publications: S805A matriptase [38], R44Q prostasin [25], C47F HAI-2 [38], R48L HAI-2 [35], C47F/R48L HAI-2 [38], R143L HAI-2, R48L/R143L HAI-2 [35], F161V HAI-2, Y163C HAI-2 and G168S HAI-2 [9]. 48 h post-transfection, cells were lysed using lysis buffer containing PBS, 1% Triton X-100 and 0.5% SDS. The cell extracts were then centrifuged for 40 min at 20.000 x g and the supernatants were stored at −20 °C.

### SDS-PAGE and Western blotting

Samples were prepared by the addition of 4 x NuPAGE^®^ LDS Sample Buffer (Invitrogen) and for reducing conditions 10 x NuPAGE^®^ Sample Reducing Agent. The samples were subsequently boiled at 100 °C for 5 min. Proteins were separated on NuPAGE^TM^ 10% Bis-Tris Midi Gels (cat.no. WG1201B0X, Invitrogen) and transferred to PVDF Transfer Membranes (Thermo Scientific). The membranes were then blocked with PBS containing 0.1% Tween-20 and 10% skimmed milk powder (10% MPBS-T) for 0.5–1 h at room temperature. Each membrane was subsequently incubated with primary antibody diluted in 1% MPBS-T at 4 °C overnight. The membranes were then washed 3 x 5 min in PBS-T and incubated with horseradish peroxidase (HRP)-conjugated secondary antibodies diluted in 1% MPBS-T for 1 h at room temperature. Following another 3 x 5 min wash in PBS-T, the membranes were developed using the WesternBright^TM^ Sirius Western blotting detection kit (Advansta) according to the protocol supplied by the manufacturer. The membranes were visualized using a Fuji LAS-1000 camera and Intelligent DarkBox II (FujiFilm Sweden AB) and the program Image Reader LAS-1000 Lite V1.5.

### Antibodies

The samples used for western blotting were all boiled and reduced. Following transfer, the membranes were incubated with one of the primary antibodies: polyclonal sheep anti-human matriptase IgG (cat.no. AF3946, R&D systems) (1:1000), polyclonal rabbit anti-human HAI-2 antibody (cat.no. HPA011101, Sigma Prestige Antibodies) (1:1000) or monoclonal mouse anti-human prostasin IgG (cat.no. 612173, BD Transduction Laboratories™) (1:1000). The membranes were subsequently incubated with the appropriate HRP-conjugated secondary antibody. For the peptidolytic activity assay, the monoclonal aZ-mAb-6 antibody [37] was used to inhibit the proteolytic activity of matriptase.

### Immunohistochemistry

24 h prior to transfection, HEK293 cells were seeded onto polylysine-coated glass coverslips in 6-well Nunclon^TM^ Cell-Culture Treated Multidishes (cat. no. 140675). For IHC, cells were transiently transfected using Lipofectamine 3000^TM^ (Invitrogen). After 48 h, the HEK293 cells were fixed in 4% formaldehyde prepared in PBS (Invitrogen) for 20 min at room temperature before being washed twice with PBS. The coverslips were then washed 3 x 5 min in wash buffer composed of Tris-buffered saline (TBS) (150 mM NaCl, 50 mM Tris-HCl, pH 7.6) with 0.01% Triton X-100. Subsequently, the coverslips were incubated in 10% goat serum for 30 min followed by 1 h incubation in primary antibody (polyclonal rabbit anti-human HAI-2 antibody) diluted 1:2000 in goat serum. The coverslips were then washed 3 x 5 min in wash buffer and incubated with HRP-conjugated secondary antibody (Dako EnVision+ System-HRP Labelled Polymer Anti-Rabbit) in goat serum for 30 min. Following another 3 x 5 min wash in wash buffer, the coverslips were incubated with 3,3′- diaminobenzidine (DAB) (EnVision) for 10 min. Subsequently, the coverslips were washed 2 x 5 min with wash buffer and 1 x 5 min with Millipore water before the cell nuclei were stained with hematoxylin (AMPQ00253, Ampliqon) for 30 sec. The coverslips were then washed in Millipore water for 5 min before being mounted onto glass slides using Aquamount (cat. no. 36086, BDH). Images were acquired on a Leica DM5500 microscope equipped with a Leica DMC6200 camera.

### Peptidolytic activity assay

Cell extracts of transiently transfected HEK293 cells were subjected to a peptidolytic activity assay. For this assay, H-d-Isoleucyl-l-prolyl-l-arginine-p-nitroaniline (cat.no. S-2288, Chromogenix) was used as a chromogenic substrate, which is cleaved by a broad spectrum of serine proteases. In order to determine the proteolytic activity of matriptase, each sample was investigated in the presence or absence of aZ-mAb-6 antibody, which specifically inhibits matriptase activity [37]. Cell extracts were treated with a similar volume of either PBS or aZ-mAb-6 antibody to a final concentration of 2 µM (corresponding to 10 x K_i_ of the aZ-mAb-6 antibody [37]). The extracts were then incubated at 37 °C for 1 h. Afterwards, 300 µM chromogenic substrate S-2288 was added to the cell lysate in an equal volume of HBS buffer composed of 20 mM HEPES (pH 7.4), 140 mM NaCl supplemented with 0.1% BSA (Sigma). The resulting color development was measured at 405 nm every min for 5 h on a standard plate reader (Biotek Synergy HT) at 37 °C. The peptidolytic activity of each sample was calculated as the rate of color development (mAU/min). Matriptase-specific activity was determined as the difference between proteolytic activity in the presence and absence of aZ-mAb-6 antibody. The aZ-mAb-6 antibody was subjected to degradation by proteases in the cell extracts. Thus, the peptidolytic assay underestimates the amount of matriptase-specific activity present in the investigated samples.

## Abbreviations

CSD: congenital sodium diarrhea
ENaC: epithelial sodium channel
GC-C: guanylate cyclase C
GnomAD: Genome Aggregation Database
HAI-1: hepatocyte growth factor activator inhibitor 1
HAI-2: hepatocyte growth factor activator inhibitor 2
HRP: horseradish peroxidase
KD: Kunitz domain
NHE3: sodium hydrogen exchanger 3
SPD: serine protease domain
TMPRSS13: transmembrane serine protease 13
