# Supplementary material for: A biochemical difference between HAI-2 variants causing syndromic and non-syndromic Congenital Sodium Diarrhea
Source: PLoS One. 2026 Sep 15;21(9):e0358119. doi: 10.1371/journal.pone.0358119 (PMC13577384; doi:10.1371/journal.pone.0358119)
Supplement: Supplementary file 1 [file pone.0358119.s002.docx]

Point-by-point answer to reviewer’s comments.

1. Is the manuscript technically sound, and do the data support the conclusions?

Reviewer #1: Yes

2. Has the statistical analysis been performed appropriately and rigorously?

Reviewer #1: No

See below

3. Have the authors made all data underlying the findings in their manuscript fully available?

Reviewer #1: Yes

4. Is the manuscript presented in an intelligible fashion and written in standard English?

Reviewer #1: Yes

5. Review Comments to the Author

Reviewer #1: This manuscript investigates the functional properties of HAI-2 variants associated with congenital sodium diarrhea, aiming to distinguish syndromic from non-syndromic forms. It provides novel insights into the biochemical characteristics of HAI-2 and addresses a rare but clinically important condition, which makes the study potentially valuable. However, I believe the manuscript requires improvements in the following points.

Thank you for this positive evaluation.

Major points

- All findings in this paper are derived solely from assays using the HEK293 cell line. It is difficult to judge whether the observed effects reflect intrinsic functions of HAI-2 or the characteristics of this particular cell line. At least for the main findings, validation should be carried out in multiple cell lines, including intestinal epithelial cells.

Serine proteases generally have a large overlap in substrate preferences and consequently it is very difficult to construct an assay to measure the enzymatic activity of these enzymes in complex solutions such as cell lysates. Because of this, generally, no specific assays are available for serine proteases. In addition, no specific inhibitors are known for these enzymes, apart from very special cases, like the inhibitory antibodies for matriptase developed by us and used in this study. The antibody makes it possible for us to specifically assay matriptase activity.

The assay using the inhibitory antibody (Figure 4) can only work if the endogenous expression of matriptase and other serine proteases with affinity for the peptide substrate is low as high endogenous expression of matriptase or endogenous serine proteases with similar specificity would likely exhaust the inhibitory capacity of the antibody and/or generate a high background. As matriptase is expressed in all epithelial cells, it is unlikely that the assay can function in any epithelial cell type. Most other types of cells express serine proteases as it is a large family of proteases abundant in many cell types. Thus, we think we were lucky to identify HEK293 cells as it does not express matriptase and has a low expression of serine proteases with affinity for our substrate, which made this assay possible.

The assay for detecting prostasin activity is based on the finding that only matriptase and prostasin are able to cleave and activate matriptase. Thus, when matriptase is mutated in its active site serine (matriptase S805A) it becomes a specific substrate for prostasin but only in cells with no expression of matriptase like HEK293 cells. Thus, also this assay cannot function in epithelial cells as they have an endogenous expression of matriptase.

See also lines 233-238

To our knowledge, the only assays described to measure matriptase and prostasin activity are these two assays developed by us and used in this study.

However, we acknowledge your concern and have now included a sentence to draw attention to this limitation of our system see lines 501-507.

- Western blot data (Fig.2A, Fig.3, Fig.5) lack loading controls. In addition, no quantification or statistical analysis is provided, so the evidence is insufficient to support the conclusions.

The assays described in the present study relies heavily on having the same transfection efficiently and the same fraction of the sample loaded. In figure 3 and 5, prostasin serve as a control for both transfection efficiency and loading.

The results in Fig.2A, Fig.3, and Fig.5 display a clear reproducible pattern. We chose to display the gels as they are, in our opinion, the western blot gave a more unbiased display of our results than quantitation due to the relative high background in some of the western blots.

Also, in Fig.2B, the evaluation method of immunostaining is unclear, and the evidence is not adequate to support the conclusion.

Thank you for this comment – we do realize that this was not explained well enough. Please see lines 195-204 and 209-216.

- HEK293 cells endogenously express HAI-2, matriptase, and prostasin, and HAI-1 as well as other serine proteases (such as hepsin) may also influence the results. The manuscript would benefit from presenting data on these endogenous expressions and including discussion on how they may have affected the results.

Even though mRNA of HAI-2, matriptase and prostasin can be detected in HEK293 cells (Vogel et al., unpublished results), post-translational mechanism causes the steady state level to be low or undetectable. In the HEK293 cells used by us there is a very low steady state level of HAI-2, which is only detectable by western blots if overexposed (Vogel et al., unpublished result). At the exposure used in this study no immunoreactivity can be detected (Figure 2 lane 1 and 10, Figure 3 lane 1 and 2, and Figure 5 lane 1 and 2).

Likewise, no prostasin immunoreactivity can be detected when prostasin is not recombinantly expressed see Figure 3, lane 1, and Figure 5 lane 1.

Also, no matriptase immunoreactivity can be detected in HEK293 lysates as previously shown by us (Nonboe *et al.*, Traffic 2017 pp378-391).

In the present paper the important parameter is not whether matriptase is expressed but whether it is activated as shown by detection of the SPD-domain. It is clear that no endogenous proteases cleave and activate matriptase in the absence of recombinant expression of prostasin, as there is a low to undetectable level of the 30 kDa SPD fragment, which is the read-out in this assay (Figure 3 lane 1 and Figure 5 lane 1).

As hepsin along with other serine proteases endogenously expressed in HEK293 cells do not cleave matriptase S805A they have no influence on the result.

Likewise, the antibody used to inhibit matriptase does not inhibit hepsin (Tamberg et al., (2018) 294(314-426)

Hepsin and other endogenous expressed serine proteases expressed in HEK293 cells are thus unlikely to affect the results described in this paper.

- For the evaluation of prostasin and matriptase activity, entirely different methods were used, which lacks consistency. The robustness of the conclusions could be strengthened by employing multiple methods, including a common approach for both proteases. If this is difficult, it would be helpful to acknowledge this inconsistency as a limitation.

Thank you for pointing this out. However, it is not possible for us to use a similar assay for matriptase and prostasin, nor is it possible to use several different assays for each protease as discussed above. However, we acknowledge your concern see lines 506-507.

- In Fig.4, HAI-2 C47F/R48L, Y68A, and Y68C appear not merely to fail to inhibit matriptase, but rather to enhance matriptase activity. The manuscript would be strengthened by explicitly discussing this observation.

Thank you for pointing this out. We have added discussion of this specific point to enhance clarity see lines 303-324.

Minor points

- The difference between Y68C and the other variants should not be overgeneralized to the difference between syndromic and non-syndromic CSD. The manuscript should be carefully re-examined from this perspective to ensure that the conclusions are phrased with appropriate caution.

Thank you for this comment. We have now rephrased the manuscript accordingly.

- From line 421 onwards, the text reads more like a Discussion, but no Discussion heading is Thank you for pointing this out. We have changed the manuscript to contain separate result and discussion sections. provided. The section structure should be reconsidered.

Thank you for pointing this out. We have changed the manuscript to contain separate result and discussion sections.

- There are several places where the logic is abrupt and the meaning difficult to follow (e.g., lines 210–214). The entire manuscript should be carefully checked to ensure a clear and coherent writing structure.

Thank you for this comment. We have checked the whole manuscript to improve clarity and coherence and deleted the text in lines 210-214.
