# Supplementary material for: A biochemical difference between HAI-2 variants causing syndromic and non-syndromic Congenital Sodium Diarrhea
Source: PLoS One. 2026 Sep 15;21(9):e0358119. doi: 10.1371/journal.pone.0358119 (PMC13577384; doi:10.1371/journal.pone.0358119)
Supplement: Supplementary file 2 [file pone.0358119.s003.docx]

PONE-D-25-38786R1

A Biochemical Difference between HAI-2 Variants causing Syndromic and Non-Syndromic Congenital Sodium Diarrhea.

Please find below a point by point response to the reviewer’s comments.

________________________________________

3. Has the statistical analysis been performed appropriately and rigorously?

**Reviewer #2: No**

Reviewer #3: Yes

**Reviewer #4: No**

***See below***

________________________________________________________________________________

5. Is the manuscript presented in an intelligible fashion and written in standard English?

PLOS ONE does not copyedit accepted manuscripts, so the language in submitted articles must be clear, correct, and unambiguous. Any typographical or grammatical errors should be corrected at revision, so please note any specific errors here.

Reviewer #2: Yes

Reviewer #3: Yes

**Reviewer #4: No**

***We are so sorry. A native English speaker has corrected typographical and grammatical errors.***

________________________________________

6. Review Comments to the Author

Reviewer #2: This manuscript addresses the functional consequences of CSD-associated HAI-2 variants and provides a mechanistic comparison between non-syndromic and syndromic forms. Overall, I find the study interesting and well motivated. The data support the conclusion that CSD-associated HAI-2 variants generally show reduced inhibition of prostasin, while the non-syndromic Y68C variant additionally displays a markedly reduced ability to inhibit matriptase. The work also offers an interesting mechanistic model suggesting that prostasin inhibition by HAI-2 may involve both Kunitz domains in a manner distinct from classical Kunitz-type inhibitors.

***Thank you for this evaluation***

This is a relatively minor point, but the statistical analysis of the functional assays would benefit from additional clarification. Although the figure legend states that differences were analyzed using a t-test with N = 3 or more, it is currently unclear what exactly N represents, and how many independent experiments were performed for each condition. Clarifying these points in the Methods and/or figure legends would improve the transparency and interpretability of the statistical analysis.

***This has been done by adding this information to the legend for Figure 4.***

Reviewer #3: Thank you for the revised submission and the point-by-point responses to previous reviewers. The revision improves clarity in several key areas, particularly by (i) explicitly acknowledging that the matriptase/prostasin activity assays are only feasible in HEK293 cells and may not generalize to intestinal epithelium, and (ii) adding statistical reporting for the peptidolytic activity assay accompanying Fig 4. The revised Results/Discussion better explains the interpretation of the activity patterns shown in Fig 4 and more cleanly separates Results and Discussion than before.

***Thank you***

Reviewer #4: The authors have addressed most of the concerns raised in the previous round and improved the manuscript. I recommend acceptance after minor revisions addressing the remaining points.

***Thank you.***

The Western blot-based conclusions remain somewhat over-stated because Fig. 2A, Fig. 3, and Fig. 5 still lack loading controls, densitometric quantification, or statistical analysis. I understand the authors’ explanation that quantification may be difficult because of background signal, and that prostasin can serve as a partial control in Fig. 3 and Fig. 5. However, claims such as “similar steady-state protein level” and “reduced ability to inhibit prostasin” should either be supported by quantification/replicate presentation, or phrased explicitly as qualitative interpretations from representative blots. At minimum, the authors should clarify in the figure legends and Results that these Western blot conclusions are based on reproducible qualitative patterns across N=3 experiments.

***Thank you for this comment. The result section and the legend to figures 2, 3 and 5 have been changed accordingly. Furthermore, the original blots from the 3 experiments in Figure 3 has been re-probed with a new (and better) lot of the original antibody used to detect matriptase and the figure has been changed accordingly and is now displaying a clearer result due to a lower background.***

For Fig. 4, the statistical reporting should be clarified. The legend states “N=3 or more,” but the exact number of biological replicates for each condition should be provided. The authors should also specify whether the t-tests were paired or unpaired, and whether any correction for multiple comparisons was applied or why it was not considered necessary.

***This information has been added to the legend for Figure 4.***

The authors have appropriately acknowledged that the assays are limited to HEK293 cells. However, some language still risks overgeneralizing from a single non-syndromic variant/case to syndromic versus non-syndromic CSD more broadly.

***We have done our best to reduce this overgeneralizing***

Several minor wording issues remain and should be corrected before publication. Examples include the sentence around line 220, where a period appears to be missing before “A proteolytically active zymogen-locked prostasin variant”; “matripase” should be “matriptase”; “I was also previously shown” should be “It was also previously shown”; and “However. it” should be “However, it.” The figure legends also contain “fugure,” which should be corrected to “figure.” Authors should do a complete screening.

***We are so sorry. A native English speaker has corrected typographical and grammatical errors. Also, the mistakes listed have been corrected.***

________________________________________
