# Supplementary material for: A biochemical difference between HAI-2 variants causing syndromic and non-syndromic Congenital Sodium Diarrhea
Source: PLoS One. 2026 Sep 15;21(9):e0358119. doi: 10.1371/journal.pone.0358119 (PMC13577384; doi:10.1371/journal.pone.0358119)
Supplement: Peer review history

# Author comment: Author response to Decision Letter 0

**DOI:** 10.1371/journal.pone.0358119.r001

**Transfer Alert**

This paper was transferred from another journal. As a result, its full editorial history (including decision letters, peer reviews and author responses) may not be present.

16 Jul 2025

# Peer review: Decision Letter 0

**DOI:** 10.1371/journal.pone.0358119.r002

**Authors:** Jianhong Zhou

16 Dec 2025

Dear Dr. Vogel,

Please submit your revised manuscript by Jan 26 2026 11:59PM. If you will need more time than this to complete your revisions, please reply to this message or contact the journal office at plosone@plos.org. When you're ready to submit your revision, log on to https://www.editorialmanager.com/pone/ and select the 'Submissions Needing Revision' folder to locate your manuscript file.

- A letter that responds to each point raised by the academic editor and reviewer(s). You should upload this letter as a separate file labeled 'Response to Reviewers'.
- A marked-up copy of your manuscript that highlights changes made to the original version. You should upload this as a separate file labeled 'Revised Manuscript with Track Changes'.
- An unmarked version of your revised paper without tracked changes. You should upload this as a separate file labeled 'Manuscript'.

If you would like to make changes to your financial disclosure, please include your updated statement in your cover letter. Guidelines for resubmitting your figure files are available below the reviewer comments at the end of this letter.

If applicable, we recommend that you deposit your laboratory protocols in protocols.io to enhance the reproducibility of your results. Protocols.io assigns your protocol its own identifier (DOI) so that it can be cited independently in the future. For instructions see: https://journals.plos.org/plosone/s/submission-guidelines#loc-laboratory-protocols. Additionally, PLOS ONE offers an option for publishing peer-reviewed Lab Protocol articles, which describe protocols hosted on protocols.io. Read more information on sharing protocols at https://plos.org/protocols?utm_medium=editorial-email&utm_source=authorletters&utm_campaign=protocols.

We look forward to receiving your revised manuscript.

Kind regards,

Jianhong Zhou

Staff Editor

PLOS One

Journal Requirements:

When submitting your revision, we need you to address these additional requirements.

1.Please ensure that your manuscript meets PLOS ONE's style requirements, including those for file naming. The PLOS ONE style templates can be found at

https://journals.plos.org/plosone/s/file?id=wjVg/PLOSOne_formatting_sample_main_body.pdf and

https://journals.plos.org/plosone/s/file?id=ba62/PLOSOne_formatting_sample_title_authors_affiliations.pdf

2. Please amend the manuscript submission data (via Edit Submission) to include author “René Villadsen”.

3. PLOS ONE now requires that authors provide the original uncropped and unadjusted images underlying all blot or gel results reported in a submission’s figures or Supporting Information files. This policy and the journal’s other requirements for blot/gel reporting and figure preparation are described in detail at https://journals.plos.org/plosone/s/figures#loc-blot-and-gel-reporting-requirements and https://journals.plos.org/plosone/s/figures#loc-preparing-figures-from-image-files. When you submit your revised manuscript, please ensure that your figures adhere fully to these guidelines and provide the original underlying images for all blot or gel data reported in your submission. See the following link for instructions on providing the original image data: https://journals.plos.org/plosone/s/figures#loc-original-images-for-blots-and-gels.

In your cover letter, please note whether your blot/gel image data are in Supporting Information or posted at a public data repository, provide the repository URL if relevant, and provide specific details as to which raw blot/gel images, if any, are not available. Email us at plosone@plos.org if you have any questions.

4. If the reviewer comments include a recommendation to cite specific previously published works, please review and evaluate these publications to determine whether they are relevant and should be cited. There is no requirement to cite these works unless the editor has indicated otherwise.

Additional Editor Comments:

Please note that we have only been able to secure a single reviewer to assess your manuscript. We are issuing a decision on your manuscript at this point to prevent further delays in the evaluation of your manuscript. Please be aware that the editor who handles your revised manuscript might find it necessary to invite additional reviewers to assess this work once the revised manuscript is submitted. However, we will aim to proceed on the basis of this single review if possible.

[Note: HTML markup is below. Please do not edit.]

Reviewer's Responses to Questions

**Comments to the Author**

1. Is the manuscript technically sound, and do the data support the conclusions?

Reviewer #1: Yes

**********

2. Has the statistical analysis been performed appropriately and rigorously?

Reviewer #1: No

**********

3. Have the authors made all data underlying the findings in their manuscript fully available?

The PLOS Data policy

Reviewer #1: Yes

**********

4. Is the manuscript presented in an intelligible fashion and written in standard English?

Reviewer #1: Yes

**********

Reviewer #1: This manuscript investigates the functional properties of HAI-2 variants associated with congenital sodium diarrhea, aiming to distinguish syndromic from non-syndromic forms. It provides novel insights into the biochemical characteristics of HAI-2 and addresses a rare but clinically important condition, which makes the study potentially valuable. However, I believe the manuscript requires improvements in the following points.

Major points

- All findings in this paper are derived solely from assays using the HEK293 cell line. It is difficult to judge whether the observed effects reflect intrinsic functions of HAI-2 or the characteristics of this particular cell line. At least for the main findings, validation should be carried out in multiple cell lines, including intestinal epithelial cells.

- Western blot data (Fig.2A, Fig.3, Fig.5) lack loading controls. In addition, no quantification or statistical analysis is provided, so the evidence is insufficient to support the conclusions. Also, in Fig.2B, the evaluation method of immunostaining is unclear, and the evidence is not adequate to support the conclusion.

- HEK293 cells endogenously express HAI-2, matriptase, and prostasin, and HAI-1 as well as other serine proteases (such as hepsin) may also influence the results. The manuscript would benefit from presenting data on these endogenous expressions and including discussion on how they may have affected the results.

- For the evaluation of prostasin and matriptase activity, entirely different methods were used, which lacks consistency. The robustness of the conclusions could be strengthened by employing multiple methods, including a common approach for both proteases. If this is difficult, it would be helpful to acknowledge this inconsistency as a limitation.

- In Fig.4, HAI-2 C47F/R48L, Y68A, and Y68C appear not merely to fail to inhibit matriptase, but rather to enhance matriptase activity. The manuscript would be strengthened by explicitly discussing this observation.

Minor points

- The difference between Y68C and the other variants should not be overgeneralized to the difference between syndromic and non-syndromic CSD. The manuscript should be carefully re-examined from this perspective to ensure that the conclusions are phrased with appropriate caution.

- From line 421 onwards, the text reads more like a Discussion, but no Discussion heading is provided. The section structure should be reconsidered.

- There are several places where the logic is abrupt and the meaning difficult to follow (e.g., lines 210–214). The entire manuscript should be carefully checked to ensure a clear and coherent writing structure.

**********

what does this mean?). If published, this will include your full peer review and any attached files.

If you choose “no”, your identity will remain anonymous but your review may still be made public.

**Do you want your identity to be public for this peer review?** For information about this choice, including consent withdrawal, please see our Privacy Policy

Reviewer #1: **Yes:** Takumi Kiwaki

**********

[NOTE: If reviewer comments were submitted as an attachment file, they will be attached to this email and accessible via the submission site. Please log into your account, locate the manuscript record, and check for the action link "View Attachments". If this link does not appear, there are no attachment files.]

To ensure your figures meet our technical requirements, please review our figure guidelines: https://journals.plos.org/plosone/s/figures

You may also use PLOS’s free figure tool, NAAS, to help you prepare publication quality figures: https://journals.plos.org/plosone/s/figures#loc-tools-for-figure-preparation.

NAAS will assess whether your figures meet our technical requirements by comparing each figure against our figure specifications.

# Author comment: Author response to Decision Letter 1

**DOI:** 10.1371/journal.pone.0358119.r003

13 Mar 2026

Point-by-point answer to reviewer’s comments.

1. Is the manuscript technically sound, and do the data support the conclusions?

Reviewer #1: Yes

2. Has the statistical analysis been performed appropriately and rigorously?

Reviewer #1: No

See below

3. Have the authors made all data underlying the findings in their manuscript fully available?

Reviewer #1: Yes

4. Is the manuscript presented in an intelligible fashion and written in standard English?

Reviewer #1: Yes

5. Review Comments to the Author

Reviewer #1: This manuscript investigates the functional properties of HAI-2 variants associated with congenital sodium diarrhea, aiming to distinguish syndromic from non-syndromic forms. It provides novel insights into the biochemical characteristics of HAI-2 and addresses a rare but clinically important condition, which makes the study potentially valuable. However, I believe the manuscript requires improvements in the following points.

Thank you for this positive evaluation.

Major points

- All findings in this paper are derived solely from assays using the HEK293 cell line. It is difficult to judge whether the observed effects reflect intrinsic functions of HAI-2 or the characteristics of this particular cell line. At least for the main findings, validation should be carried out in multiple cell lines, including intestinal epithelial cells.

Serine proteases generally have a large overlap in substrate preferences and consequently it is very difficult to construct an assay to measure the enzymatic activity of these enzymes in complex solutions such as cell lysates. Because of this, generally, no specific assays are available for serine proteases. In addition, no specific inhibitors are known for these enzymes, apart from very special cases, like the inhibitory antibodies for matriptase developed by us and used in this study. The antibody makes it possible for us to specifically assay matriptase activity.

The assay using the inhibitory antibody (Figure 4) can only work if the endogenous expression of matriptase and other serine proteases with affinity for the peptide substrate is low as high endogenous expression of matriptase or endogenous serine proteases with similar specificity would likely exhaust the inhibitory capacity of the antibody and/or generate a high background. As matriptase is expressed in all epithelial cells, it is unlikely that the assay can function in any epithelial cell type. Most other types of cells express serine proteases as it is a large family of proteases abundant in many cell types. Thus, we think we were lucky to identify HEK293 cells as it does not express matriptase and has a low expression of serine proteases with affinity for our substrate, which made this assay possible.

The assay for detecting prostasin activity is based on the finding that only matriptase and prostasin are able to cleave and activate matriptase. Thus, when matriptase is mutated in its active site serine (matriptase S805A) it becomes a specific substrate for prostasin but only in cells with no expression of matriptase like HEK293 cells. Thus, also this assay cannot function in epithelial cells as they have an endogenous expression of matriptase.

See also lines 233-238

To our knowledge, the only assays described to measure matriptase and prostasin activity are these two assays developed by us and used in this study.

However, we acknowledge your concern and have now included a sentence to draw attention to this limitation of our system see lines 501-507.

- Western blot data (Fig.2A, Fig.3, Fig.5) lack loading controls. In addition, no quantification or statistical analysis is provided, so the evidence is insufficient to support the conclusions.

The assays described in the present study relies heavily on having the same transfection efficiently and the same fraction of the sample loaded. In figure 3 and 5, prostasin serve as a control for both transfection efficiency and loading.

The results in Fig.2A, Fig.3, and Fig.5 display a clear reproducible pattern. We chose to display the gels as they are, in our opinion, the western blot gave a more unbiased display of our results than quantitation due to the relative high background in some of the western blots.

Also, in Fig.2B, the evaluation method of immunostaining is unclear, and the evidence is not adequate to support the conclusion.

Thank you for this comment – we do realize that this was not explained well enough. Please see lines 195-204 and 209-216.

- HEK293 cells endogenously express HAI-2, matriptase, and prostasin, and HAI-1 as well as other serine proteases (such as hepsin) may also influence the results. The manuscript would benefit from presenting data on these endogenous expressions and including discussion on how they may have affected the results.

Even though mRNA of HAI-2, matriptase and prostasin can be detected in HEK293 cells (Vogel et al., unpublished results), post-translational mechanism causes the steady state level to be low or undetectable. In the HEK293 cells used by us there is a very low steady state level of HAI-2, which is only detectable by western blots if overexposed (Vogel et al., unpublished result). At the exposure used in this study no immunoreactivity can be detected (Figure 2 lane 1 and 10, Figure 3 lane 1 and 2, and Figure 5 lane 1 and 2).

Likewise, no prostasin immunoreactivity can be detected when prostasin is not recombinantly expressed see Figure 3, lane 1, and Figure 5 lane 1.

Also, no matriptase immunoreactivity can be detected in HEK293 lysates as previously shown by us (Nonboe et al., Traffic 2017 pp378-391).

In the present paper the important parameter is not whether matriptase is expressed but whether it is activated as shown by detection of the SPD-domain. It is clear that no endogenous proteases cleave and activate matriptase in the absence of recombinant expression of prostasin, as there is a low to undetectable level of the 30 kDa SPD fragment, which is the read-out in this assay (Figure 3 lane 1 and Figure 5 lane 1).

As hepsin along with other serine proteases endogenously expressed in HEK293 cells do not cleave matriptase S805A they have no influence on the result.

Likewise, the antibody used to inhibit matriptase does not inhibit hepsin (Tamberg et al., (2018) 294(314-426)

Hepsin and other endogenous expressed serine proteases expressed in HEK293 cells are thus unlikely to affect the results described in this paper.

- For the evaluation of prostasin and matriptase activity, entirely different methods were used, which lacks consistency. The robustness of the conclusions could be strengthened by employing multiple methods, including a common approach for both proteases. If this is difficult, it would be helpful to acknowledge this inconsistency as a limitation.

Thank you for pointing this out. However, it is not possible for us to use a similar assay for matriptase and prostasin, nor is it possible to use several different assays for each protease as discussed above. However, we acknowledge your concern see lines 506-507.

- In Fig.4, HAI-2 C47F/R48L, Y68A, and Y68C appear not merely to fail to inhibit matriptase, but rather to enhance matriptase activity. The manuscript would be strengthened by explicitly discussing this observation.

Thank you for pointing this out. We have added discussion of this specific point to enhance clarity see lines 303-324.

Minor points

- The difference between Y68C and the other variants should not be overgeneralized to the difference between syndromic and non-syndromic CSD. The manuscript should be carefully re-examined from this perspective to ensure that the conclusions are phrased with appropriate caution.

Thank you for this comment. We have now rephrased the manuscript accordingly.

- From line 421 onwards, the text reads more like a Discussion, but no Discussion heading is Thank you for pointing this out. We have changed the manuscript to contain separate result and discussion sections. provided. The section structure should be reconsidered.

Thank you for pointing this out. We have changed the manuscript to contain separate result and discussion sections.

- There are several places where the logic is abrupt and the meaning difficult to follow (e.g., lines 210–214). The entire manuscript should be carefully checked to ensure a clear and coherent writing structure.

Thank you for this comment. We have checked the whole manuscript to improve clarity and coherence and deleted the text in lines 210-214.

Attachment Submitted filename: Response to reviewers.docx

# Peer review: Decision Letter 1

**DOI:** 10.1371/journal.pone.0358119.r004

**Authors:** Yun Li

11 May 2026

Dear Dr. Vogel,

Please submit your revised manuscript by Jun 25 2026 11:59PM. If you will need more time than this to complete your revisions, please reply to this message or contact the journal office at plosone@plos.org. When you're ready to submit your revision, log on to https://www.editorialmanager.com/pone/ and select the 'Submissions Needing Revision' folder to locate your manuscript file.

- A letter that responds to each point raised by the academic editor and reviewer(s). You should upload this letter as a separate file labeled 'Response to Reviewers'.
- A marked-up copy of your manuscript that highlights changes made to the original version. You should upload this as a separate file labeled 'Revised Manuscript with Track Changes'.
- An unmarked version of your revised paper without tracked changes. You should upload this as a separate file labeled 'Manuscript'.

If you would like to make changes to your financial disclosure, please include your updated statement in your cover letter. Guidelines for resubmitting your figure files are available below the reviewer comments at the end of this letter.

If applicable, we recommend that you deposit your laboratory protocols in protocols.io to enhance the reproducibility of your results. Protocols.io assigns your protocol its own identifier (DOI) so that it can be cited independently in the future. For instructions see: https://journals.plos.org/plosone/s/submission-guidelines#loc-laboratory-protocols. Additionally, PLOS ONE offers an option for publishing peer-reviewed Lab Protocol articles, which describe protocols hosted on protocols.io. Read more information on sharing protocols at https://plos.org/protocols?utm_medium=editorial-email&utm_source=authorletters&utm_campaign=protocols.

As the corresponding author, your ORCID iD is verified in the submission system and will appear in the published article. PLOS supports the use of ORCID, and we encourage all coauthors to register for an ORCID iD and use it as well. Please encourage your coauthors to verify their ORCID iD within the submission system before final acceptance, as unverified ORCID iDs will not appear in the published article. *Only* the individual author can complete the verification step; PLOS staff *cannot* verify ORCID iDs on behalf of authors.

We look forward to receiving your revised manuscript.

Kind regards,

Yun Li

Academic Editor

PLOS One

Journal Requirements:

If the reviewer comments include a recommendation to cite specific previously published works, please review and evaluate these publications to determine whether they are relevant and should be cited. There is no requirement to cite these works unless the editor has indicated otherwise.

Please review your reference list to ensure that it is complete and correct. If you have cited papers that have been retracted, please include the rationale for doing so in the manuscript text, or remove these references and replace them with relevant current references. Any changes to the reference list should be mentioned in the rebuttal letter that accompanies your revised manuscript. If you need to cite a retracted article, indicate the article’s retracted status in the References list and also include a citation and full reference for the retraction notice.

[Note: HTML markup is below. Please do not edit.]

Reviewers' comments:

Reviewer's Responses to Questions

**Comments to the Author**

Reviewer #2: (No Response)

Reviewer #3: All comments have been addressed

Reviewer #4: (No Response)

**********

2. Is the manuscript technically sound, and do the data support the conclusions?

Reviewer #2: Yes

Reviewer #3: Yes

Reviewer #4: Yes

**********

3. Has the statistical analysis been performed appropriately and rigorously?

Reviewer #2: No

Reviewer #3: Yes

Reviewer #4: No

**********

4. Have the authors made all data underlying the findings in their manuscript fully available?

The PLOS Data policy

Reviewer #2: Yes

Reviewer #3: Yes

Reviewer #4: Yes

**********

5. Is the manuscript presented in an intelligible fashion and written in standard English?

Reviewer #2: Yes

Reviewer #3: Yes

Reviewer #4: No

**********

Reviewer #2: This manuscript addresses the functional consequences of CSD-associated HAI-2 variants and provides a mechanistic comparison between non-syndromic and syndromic forms. Overall, I find the study interesting and well motivated. The data support the conclusion that CSD-associated HAI-2 variants generally show reduced inhibition of prostasin, while the non-syndromic Y68C variant additionally displays a markedly reduced ability to inhibit matriptase. The work also offers an interesting mechanistic model suggesting that prostasin inhibition by HAI-2 may involve both Kunitz domains in a manner distinct from classical Kunitz-type inhibitors.

This is a relatively minor point, but the statistical analysis of the functional assays would benefit from additional clarification. Although the figure legend states that differences were analyzed using a t-test with N = 3 or more, it is currently unclear what exactly N represents, and how many independent experiments were performed for each condition. Clarifying these points in the Methods and/or figure legends would improve the transparency and interpretability of the statistical analysis.

Reviewer #3: Thank you for the revised submission and the point-by-point responses to previous reviewers. The revision improves clarity in several key areas, particularly by (i) explicitly acknowledging that the matriptase/prostasin activity assays are only feasible in HEK293 cells and may not generalize to intestinal epithelium, and (ii) adding statistical reporting for the peptidolytic activity assay accompanying Fig 4. The revised Results/Discussion better explains the interpretation of the activity patterns shown in Fig 4 and more cleanly separates Results and Discussion than before.

Reviewer #4: The authors have addressed most of the concerns raised in the previous round and improved the manuscript. I recommend acceptance after minor revisions addressing the remaining points.

The Western blot-based conclusions remain somewhat over-stated because Fig. 2A, Fig. 3, and Fig. 5 still lack loading controls, densitometric quantification, or statistical analysis. I understand the authors’ explanation that quantification may be difficult because of background signal, and that prostasin can serve as a partial control in Fig. 3 and Fig. 5. However, claims such as “similar steady-state protein level” and “reduced ability to inhibit prostasin” should either be supported by quantification/replicate presentation, or phrased explicitly as qualitative interpretations from representative blots. At minimum, the authors should clarify in the figure legends and Results that these Western blot conclusions are based on reproducible qualitative patterns across N=3 experiments.

For Fig. 4, the statistical reporting should be clarified. The legend states “N=3 or more,” but the exact number of biological replicates for each condition should be provided. The authors should also specify whether the t-tests were paired or unpaired, and whether any correction for multiple comparisons was applied or why it was not considered necessary.

The authors have appropriately acknowledged that the assays are limited to HEK293 cells. However, some language still risks overgeneralizing from a single non-syndromic variant/case to syndromic versus non-syndromic CSD more broadly.

Several minor wording issues remain and should be corrected before publication. Examples include the sentence around line 220, where a period appears to be missing before “A proteolytically active zymogen-locked prostasin variant”; “matripase” should be “matriptase”; “I was also previously shown” should be “It was also previously shown”; and “However. it” should be “However, it.” The figure legends also contain “fugure,” which should be corrected to “figure.” Authors should do a complete screening.

**********

what does this mean?). If published, this will include your full peer review and any attached files.

If you choose “no”, your identity will remain anonymous but your review may still be made public.

**Do you want your identity to be public for this peer review?** For information about this choice, including consent withdrawal, please see our Privacy Policy

Reviewer #2: No

Reviewer #3: No

Reviewer #4: No

**********

[NOTE: If reviewer comments were submitted as an attachment file, they will be attached to this email and accessible via the submission site. Please log into your account, locate the manuscript record, and check for the action link "View Attachments". If this link does not appear, there are no attachment files.]

To ensure your figures meet our technical requirements, please review our figure guidelines: https://journals.plos.org/plosone/s/figures

You may also use PLOS’s free figure tool, NAAS, to help you prepare publication quality figures: https://journals.plos.org/plosone/s/figures#loc-tools-for-figure-preparation.

NAAS will assess whether your figures meet our technical requirements by comparing each figure against our figure specifications.

# Author comment: Author response to Decision Letter 2

**DOI:** 10.1371/journal.pone.0358119.r005

23 Jul 2026

PONE-D-25-38786R1

A Biochemical Difference between HAI-2 Variants causing Syndromic and Non-Syndromic Congenital Sodium Diarrhea.

Please find below a point by point response to the reviewer’s comments.

________________________________________

3. Has the statistical analysis been performed appropriately and rigorously?

Reviewer #2: No

Reviewer #3: Yes

Reviewer #4: No

See below

________________________________________________________________________________

5. Is the manuscript presented in an intelligible fashion and written in standard English?

PLOS ONE does not copyedit accepted manuscripts, so the language in submitted articles must be clear, correct, and unambiguous. Any typographical or grammatical errors should be corrected at revision, so please note any specific errors here.

Reviewer #2: Yes

Reviewer #3: Yes

Reviewer #4: No

We are so sorry. A native English speaker has corrected typographical and grammatical errors.

________________________________________

6. Review Comments to the Author

Reviewer #2: This manuscript addresses the functional consequences of CSD-associated HAI-2 variants and provides a mechanistic comparison between non-syndromic and syndromic forms. Overall, I find the study interesting and well motivated. The data support the conclusion that CSD-associated HAI-2 variants generally show reduced inhibition of prostasin, while the non-syndromic Y68C variant additionally displays a markedly reduced ability to inhibit matriptase. The work also offers an interesting mechanistic model suggesting that prostasin inhibition by HAI-2 may involve both Kunitz domains in a manner distinct from classical Kunitz-type inhibitors.

Thank you for this evaluation

This is a relatively minor point, but the statistical analysis of the functional assays would benefit from additional clarification. Although the figure legend states that differences were analyzed using a t-test with N = 3 or more, it is currently unclear what exactly N represents, and how many independent experiments were performed for each condition. Clarifying these points in the Methods and/or figure legends would improve the transparency and interpretability of the statistical analysis.

This has been done by adding this information to the legend for Figure 4.

Reviewer #3: Thank you for the revised submission and the point-by-point responses to previous reviewers. The revision improves clarity in several key areas, particularly by (i) explicitly acknowledging that the matriptase/prostasin activity assays are only feasible in HEK293 cells and may not generalize to intestinal epithelium, and (ii) adding statistical reporting for the peptidolytic activity assay accompanying Fig 4. The revised Results/Discussion better explains the interpretation of the activity patterns shown in Fig 4 and more cleanly separates Results and Discussion than before.

Thank you

Reviewer #4: The authors have addressed most of the concerns raised in the previous round and improved the manuscript. I recommend acceptance after minor revisions addressing the remaining points.

Thank you.

The Western blot-based conclusions remain somewhat over-stated because Fig. 2A, Fig. 3, and Fig. 5 still lack loading controls, densitometric quantification, or statistical analysis. I understand the authors’ explanation that quantification may be difficult because of background signal, and that prostasin can serve as a partial control in Fig. 3 and Fig. 5. However, claims such as “similar steady-state protein level” and “reduced ability to inhibit prostasin” should either be supported by quantification/replicate presentation, or phrased explicitly as qualitative interpretations from representative blots. At minimum, the authors should clarify in the figure legends and Results that these Western blot conclusions are based on reproducible qualitative patterns across N=3 experiments.

Thank you for this comment. The result section and the legend to figures 2, 3 and 5 have been changed accordingly. Furthermore, the original blots from the 3 experiments in Figure 3 has been re-probed with a new (and better) lot of the original antibody used to detect matriptase and the figure has been changed accordingly and is now displaying a clearer result due to a lower background.

For Fig. 4, the statistical reporting should be clarified. The legend states “N=3 or more,” but the exact number of biological replicates for each condition should be provided. The authors should also specify whether the t-tests were paired or unpaired, and whether any correction for multiple comparisons was applied or why it was not considered necessary.

This information has been added to the legend for Figure 4.

The authors have appropriately acknowledged that the assays are limited to HEK293 cells. However, some language still risks overgeneralizing from a single non-syndromic variant/case to syndromic versus non-syndromic CSD more broadly.

We have done our best to reduce this overgeneralizing

Several minor wording issues remain and should be corrected before publication. Examples include the sentence around line 220, where a period appears to be missing before “A proteolytically active zymogen-locked prostasin variant”; “matripase” should be “matriptase”; “I was also previously shown” should be “It was also previously shown”; and “However. it” should be “However, it.” The figure legends also contain “fugure,” which should be corrected to “figure.” Authors should do a complete screening.

We are so sorry. A native English speaker has corrected typographical and grammatical errors. Also, the mistakes listed have been corrected.

________________________________________

________________________________________

Attachment Submitted filename: Response_to_Reviewers_auresp_2.docx

# Peer review: Decision Letter 2

**DOI:** 10.1371/journal.pone.0358119.r006

**Authors:** Patrick Goymer

27 Aug 2026

A Biochemical Difference between HAI-2 Variants causing Syndromic and Non-Syndromic Congenital Sodium Diarrhea.

PONE-D-25-38786R2

Dear Dr. Vogel,

We’re pleased to inform you that your manuscript has been judged scientifically suitable for publication and will be formally accepted for publication once it meets all outstanding technical requirements.

Within one week, you’ll receive an e-mail detailing the required amendments. When these have been addressed, you’ll receive a formal acceptance letter and your manuscript will be scheduled for publication.

An invoice will be generated when your article is formally accepted. Please note, if your institution has a publishing partnership with PLOS and your article meets the relevant criteria, all or part of your publication costs will be covered. Please make sure your user information is up-to-date by logging into Editorial Manager at Editorial Manager® and clicking the ‘Update My Information' link at the top of the page. For questions related to billing, please contact billing support.

If your institution or institutions have a press office, please notify them about your upcoming paper to help maximize its impact. If they’ll be preparing press materials, please inform our press team as soon as possible -- no later than 48 hours after receiving the formal acceptance. Your manuscript will remain under strict press embargo until 2 pm Eastern Time on the date of publication. For more information, please contact onepress@plos.org.

Kind regards,

Patrick Goymer

Staff Editor

PLOS One

Additional Editor Comments (optional):

Reviewers' comments:

Reviewer's Responses to Questions

**Comments to the Author**

Reviewer #4: All comments have been addressed

**********

2. Is the manuscript technically sound, and do the data support the conclusions?

Reviewer #4: Yes

**********

3. Has the statistical analysis been performed appropriately and rigorously?

Reviewer #4: Yes

**********

4. Have the authors made all data underlying the findings in their manuscript fully available?

The PLOS Data policy

Reviewer #4: Yes

**********

5. Is the manuscript presented in an intelligible fashion and written in standard English?

Reviewer #4: Yes

**********

Reviewer #4: (No Response)

**********

what does this mean?). If published, this will include your full peer review and any attached files.

If you choose “no”, your identity will remain anonymous but your review may still be made public.

**Do you want your identity to be public for this peer review?** For information about this choice, including consent withdrawal, please see our Privacy Policy

Reviewer #4: No

**********

# Editor report: Acceptance letter

**DOI:** 10.1371/journal.pone.0358119.r007

**Authors:** Patrick Goymer

PONE-D-25-38786R2

PLOS One

Dear Dr. Vogel,

I'm pleased to inform you that your manuscript has been deemed suitable for publication in PLOS One. Congratulations! Your manuscript is now being handed over to our production team.

At this stage, our production department will prepare your paper for publication. This includes ensuring the following:

* All references, tables, and figures are properly cited

* All relevant supporting information is included in the manuscript submission,

* There are no issues that prevent the paper from being properly typeset

You will receive further instructions from the production team, including instructions on how to review your proof when it is ready. Please keep in mind that we are working through a large volume of accepted articles, so please give us a few days to review your paper and let you know the next and final steps.

Lastly, if your institution or institutions have a press office, please let them know about your upcoming paper now to help maximize its impact. If they'll be preparing press materials, please inform our press team within the next 48 hours. Your manuscript will remain under strict press embargo until 2 pm Eastern Time on the date of publication. For more information, please contact onepress@plos.org.

You will receive an invoice from PLOS for your publication fee after your manuscript has reached the completed accept phase. If you receive an email requesting payment before acceptance or for any other service, this may be a phishing scheme. Learn how to identify phishing emails and protect your accounts at https://explore.plos.org/phishing.

If we can help with anything else, please email us at customercare@plos.org.

Thank you for submitting your work to PLOS One and supporting open access.

Kind regards,

PLOS One Editorial Office Staff

on behalf of

Dr Patrick Goymer

Staff Editor

PLOS One
